# Recent Advances in Camel Immunology

**DOI:** 10.3389/fimmu.2020.614150

**Published:** 2021-01-25

**Authors:** Jamal Hussen, Hans-Joachim Schuberth

**Affiliations:** ^1^ Department of Microbiology, College of Veterinary Medicine, King Faisal University, Al-Ahsa, Saudi Arabia; ^2^ Institute of Immunology, University of Veterinary Medicine Hannover, Foundation, Hannover, Germany

**Keywords:** camel (*Camelus dromedarius*), immune, overview, review—systematic, leukocytes, monocyte subpopulations

## Abstract

Camels are domesticated animals that are highly adapted to the extreme desert ecosystem with relatively higher resistance to a wide range of pathogens compared to many other species from the same geographical region. Recently, there has been increased interest in the field of camel immunology. As the progress in the analysis of camel immunoglobulins has previously been covered in many recent reviews, this review intends to summarize published findings related to camel cellular immunology with a focus on the phenotype and functionality of camel leukocyte subpopulations. The review also describes the impact of different physiological (age and pregnancy) and pathological (e.g. infection) conditions on camel immune cells. Despite the progress achieved in the field of camel immunology, there are gaps in our complete understanding of the camel immune system. Questions remain regarding innate recognition mechanisms, the functional characterization of antigen-presenting cells, and the characterization of camel NK and cytotoxic T cells.

## Introduction

Camels (*Camelus* spp.) are essential inhabitants of desert and semi-desert ecosystems (1). Unlike many other domestic species, camels thrive despite extreme temperatures, scarce vegetation, and very limited food and water resources (2, 3). The family of Camelidae comprises two major subfamilies, namely Camelinae (Old World camelids) and Laminae (New World camelids). The Old World camelids include two domesticated species: the dromedary or one humped camel (*Camelus dromedarius*) and the two humped camel or Bactrian camel (*Camelus bactrianus*) (4, 5). The wild camel (*Camelus ferus*) is a third species of Old World camelids, which is a double-humped camel living in central Asia and closely related to the Bactrian camel (6). The New World camelids, which live in the high altitudes of South America, comprise four main species including two wild species (guanaco and vicuña) and two domesticated species (llama and alpaca) (7).

In addition to their economic importance as domestic food animals in many regions of the world including the Middle East, different parts of Africa, and most regions of Asia (8, 9), camels are also found in circus or zoological collections in the northern hemisphere (10). Camels are of zoonotic importance due to many pathogens that can be transmitted to humans. For example, dromedary camels are considered as the main reservoir for the lethal zoonotic coronavirus, which is responsible for Middle East Respiratory Syndrome (MERS) in humans (11).

The immune system consists of a complex network of cellular and non-cellular components, which contribute equally to effective immune responses against pathogens. Whereas considerable research has been devoted to studying camel immunoglobulins (12), rather less attention has been paid to the cellular compartment of the camel immune system. As the progress achieved in the analysis of camel immunoglobulins has previously been covered in many recent reviews (5, 12–14), the present review will highlight the most important findings concerning camel cellular immunology. The review will especially focus on recent phenotyping and functional studies characterizing camel blood leukocyte subpopulations. In addition, the impact of different physiological (age and pregnancy) and pathological (e.g. infection) conditions on the cellular immune compartment will be discussed.

In comparison to other species from the same geographical area, camels show higher resistance to some infectious diseases and environmental stress (15–19). Compared to the severe course of many Middle East Respiratory Syndrome Coronavirus (MERS-CoV) infections in humans, camels show only mild and transient respiratory symptoms with no need for veterinary care (15, 16). Possible mechanisms for the higher resistance of camels to MERS-CoV are discussed below (*The camel immune response to MERS-CoV*). Dromedary camels also appeared to be resistant to infectious doses of foot-and-mouth disease virus (FMDV), which were sufficient to infect sheep in the same experiment (18). This type of resistance is species-dependent, since FMDV is more infectious for Bactrian than for dromedary camels (18). FMDV utilizes different integrin heterodimers (αvβ1, αvβ3, αvβ6, and αvβ8) as cellular receptors (20). Whether these integrins are differentially expressed or regulated in the susceptible and resistant species, and whether the higher resistance of camels to FMDV is determined at the level of virus-integrin interaction, represents an important question that has yet to be addressed. Dromedary camels are also well adapted to extreme levels of heat stress (19). Compared to humans, this may rely on a faster, stronger and differential induction of heat-shock protein family members and the higher resistance of general protein synthesis in response to thermal stress (21, 22). Deeper insights into the mechanisms behind the higher resistance of camels to some infectious agents and the adaptation of camel immune cells towards thermal stress are still pending.

Several immunogenomic studies described the genomic diversity of immunity-related genes in domesticated and wild camels, including genes encoding for B cell receptors, T cell receptors, and MHC molecules (6, 23–29).

## The Leukogram Pattern of Camels in Health and Disease

The species-specific leukogram, which comprises the total white blood cell (WBC) count and the relative proportions of the main leukocyte subpopulation including neutrophils, eosinophils, basophils, lymphocytes, and monocytes, provides a cost-effective evaluation tool in human and veterinary medicine, supporting disease diagnosis and guiding therapy and prognosis prediction.

A broad total WBC count range in the healthy dromedary camel, from 8.3 to 19.6 cell x10^3^/µl blood, has been reported in the literature (30, 31). Lower (32) as well as higher (33) WBC counts were reported for the Bactrian camel in comparison to the dromedary camel. In general, camels have a higher WBC count than domestic ruminants (33, 34). This is mainly due to higher numbers of neutrophils in camel blood. The fraction of neutrophils among blood leukocytes accounts for up to 77% followed by lymphocytes (30% on average) (32, 35–38). This is in contrast to domestic ruminants, where lymphocytes outnumber the leukocyte population in blood (33). The dominance of neutrophils among camel blood leukocytes results on average in a very high neutrophils to lymphocyte ratio (NLR) of 5:1 compared to a NLR of 1:2 found in domestic ruminants (39, 40). The NLR is a novel marker which has been found to be associated with systemic inflammatory responses (41–44). In other species, high NLR has been linked to impaired immune cell function and was indicative of poor patient survival in different diseases including sepsis (45, 46) and autoimmune diseases (47). The clinical relevance of the relatively high NLR in camels and its impact on the functionality of the camel immune system still needs to be investigated.

Historically, there has been a great deal of discrepency in the literature regarding leukocyte composition in camels, depending on the techniques used to analyze the samples. Earlier studies mostly applied hemocytometers to estimate the camel leukogram with settings adapted from other species. According to those studies, lymphocytes represent the most abundant leukocyte subpopulation in camel blood followed by neutrophils (48–54). However, recent hemocytometer and flow cytometric studies (55–57) identified neutrophils as the main fraction of camel leukocytes (36, 58–62).

Numerous studies investigated the changes in the camel leukogram pattern under different physiologic (age, sex, pregnancy) (63–65) and pathologic (infection) conditions (66, 67). As most camel leukogram studies presented leukocyte composition as relative values (fractions) rather than absolute values, it is difficult to compare the results obtained from different studies.

### Impact of Animal Age and Sex on the Camel Leukogram

The impact of age on the camel leukogram has been described in different studies (35, 68). In comparison to adults, young camels show higher WBC counts with higher percentages of lymphocytes and eosinophilic granulocytes but lower percentage of neutrophilic granulocytes (35). The leukogram pattern of the newborn camel calf is discussed below in detail.

Although some studies reported higher WBC counts in male camels (69), in general the animal sex shows no impact on total WBC count or differential leukocyte composition (32, 70, 71). Some authors reported a higher proportion of lymphocytes among leukocytes in males compared to female camels (70, 72), while the eosinophil fraction was higher in females compared to males (70). The impact of pregnancy on the camel leukogram (60) and the detailed immunophenotype of leukocytes in pregnant she-camels will be discussed in a separate section below.

### Seasonal Effects on the Camel Leukogram

There have been inconsistent observations regarding the impact of seasonal effects on the camel leukogram (70, 72, 73). For instance, according to Mehrotra and Gupta (74), the number of leukocytes tends to decline during the summer season. In contrast, Babeker et al. reported higher numbers of WBC with increased total numbers of neutrophils, lymphocytes, eosinophils, and basophils in camel blood during the summer season in comparison to the winter (73).

### The Camel Leukogram Pattern During Infectious Diseases

Changes in leukocytes count and composition during different parasitic and bacterial infections have been frequently described. For example, trypanosomiasis in camels induces a marked increase in the number of WBC with increased percentages of neutrophils and reduced percentages of lymphocytes (49). Relative eosinophilia (elevated fraction of eosinophils in blood) has been reported in camels suffering from different parasitic infestations, including Trypanosoma evansi (75), gastrointestinal helminths (71), and nasal Cephalopina titillator (76). In camels infected with the blood parasite Theileria anulata, the leukogram pattern was characterized by leukocytosis, neutrophilia, eosinophilia, and lymphopenia (67). Bacterial pathogens are mainly responsible for post-partal infections in camels, including mastitis and metritis (77–80). The leukogram pattern associated with camel endometritis has been described for the clinical and subclinical form of the disease. Clinical endometritis in camels is characterized by a significant rise in the total cell count of blood leukocytes, which is mainly due to higher cell numbers of neutrophils (37, 81). However, in the case of subclinical endometritis she-camels did not show a different leukogram in comparison to healthy animals (82).

## Characterization of Phenotype and Function of Camel Leukocyte Subpopulations

Early attempts to study the cellular immune system in camels were hampered by the limited availability of camel-specific monoclonal antibodies (83, 84). As the production of monoclonal antibodies is a very costly process, attempts have been made to evaluate the cross-reactivity of commercially available monoclonal antibodies raised against leukocyte antigens of ruminants, swine, or human, with camel leukocyte antigens. Using the identified cross-reactive antibodies (**Table 1**) and flow cytometry, several camel myeloid and lymphoid immune cell populations and subpopulations have been recently characterized (59, 83–85, 87–89). The antibody toolbox for camel leukocyte antigens includes antibodies to several myeloid markers such as CD172a, CD14, and CD163, and major histocompatibility complex (MHC) class I and II molecules. Using those monoclonal antibodies in combination with antibodies against cell adhesion molecules (CD11a, CD11b, CD18, and CD62L) has enabled the characterization of camel monocyte subsets. Monoclonal antibodies specific for CD4 and WC1 molecules allowed for the chracterization of camel CD4-positive T cells and γδ-T cells. The characterization of other important lymphocyte subpopulation, especially CD8-positive T cells and NK cells, still requires the identification of monoclonal antibodies to camel CD8 and CD335 (NKp46) molecules.

**Table 1:** Camel leukocyte antigen cross-reactive monoclonal antibodies.

Antigen	Clone	Isotype	Source
CD4	GC50A1	mIgM	WSU
CD11a	G43-25B	mIgG2a	BD
CD11a	HUH73A	mIgG1	WSU
CD11b	ICRF44	mIgG1	BD
CD14	TÜK4	mIgG2a	Biorad
CD14	M5E2	mIgG2a	BD
CD14	CAM36A	mIgG1	WSU
CD18	6.7	mIgG1	BD
CD18	HUH82A	mIgG2a	WSU
CD26	polyclonal	gIgG	R&D systems
CD44	LT41A	mIgG2a	WSU
CD45	LT12A	mIgG2a	WSU
CD62L	MEL14	mIgG2a	Biolegend
CD163	LND68A	mIgG1	Kingfisher
CD172a	DH59b	mIgG1	WSU
B cells	GC26A	mIgM	WSU
MHCI	H58A	mIgG2a	WSU
MHCII	TH81A5	mIgG2a	WSU
MHCII	TH14B	mIgG2a	WSU
MHCII	L243	mIgG2a	BD
WC1	BAQ128A	mIgG1	Kingfisher
WC1N2	BAQ4A	mIgG1	WSU
Activation marker	LH9A	mIgM	WSU
Activation marker	VPM30	mIgM	Biorad

MHC, Major Histocompatibility Complex; WSU, Washington State University; BD, Becton Dickinson; mIgM, mouse immunoglobulin M; mIgG, mouse immunoglobulin G; gIgG, goat IgG (36, 59, 61, 85, 86).

### Camel Neutrophilic Granulocytes

Flow cytometric analyses identified camel blood neutrophils as highly complex/granular cells (side scatter, SSC^high^) expressing CD45 and CD172a (35, 62). The higher green autofluorescence of eosinophils can be used to differentiate between camel eosinophils and neutrophils within the granulocyte population (35, 60).

Neutrophil recruitment is a cascade process organized by a set of cell adhesion molecules, which mediate their adhesion to endothelial cells of blood vessels and the subsequent steps of extravasation (90). Compared with human neutrophils, dromedary camel neutrophils express similar levels of the integrins LFA1 (CD11b/CD18) and MAC1 (also known as αMβ2; CD11b/CD18) (59).

**Figure 1:**
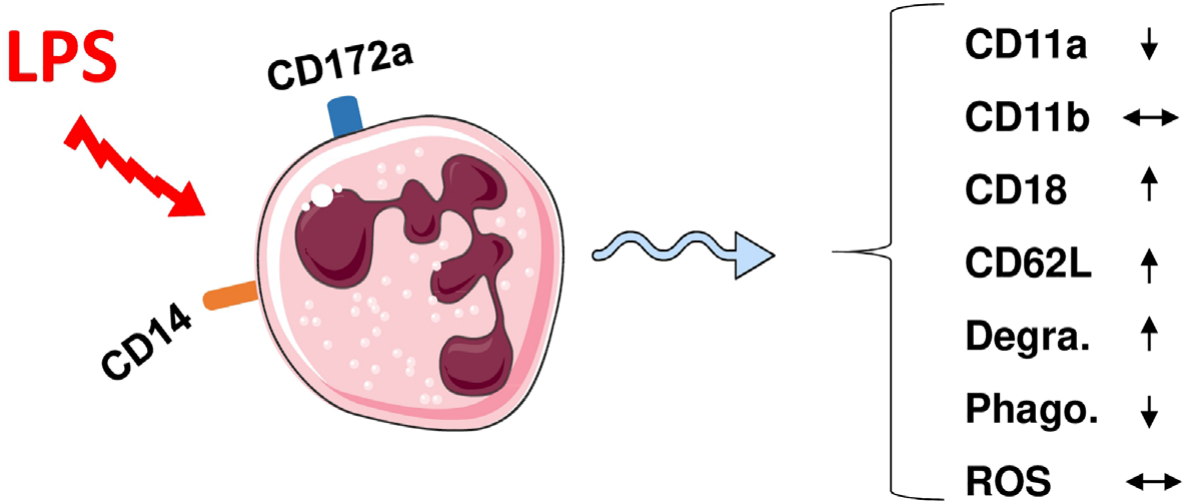
Modulation of phenotype and function of camel neutrophils by LPS stimulation. Camel neutrophils can be identified as CD172a^high^SSC^high^ leukocytes (CD45+) with low expression of CD14. Stimulation of whole camel blood with LPS induces the modulation of the expression pattern of different adhesion molecules and different antimicrobial functions (reduced SSC values as an indicator for neutrophil granularity, decreased phagocytosis capacity but no change in ROS production). Degra, Degranulation; Phago, phagocytosis; ROS, generation of reactive oxygen species.

Similar to bovine neutrophils (91), camel neutrophils show a low but distinct expression level of the LPS co-receptor CD14, suggesting a role in the sensing of gram-negative bacteria (35). This has been partially proven in whole blood stimulation assays (**Figure 1**) where LPS induced the activation and degranulation of camel neutrophils. In addition, LPS stimulation reduced the phagocytosis activity of camel neutrophils, while their ROS generating potential remained unchanged (62).

A series of recent studies on human neutrophils has indicated that distinct neutrophil subsets exist within the whole neutrophil population with diverse roles in infection and inflammation (92–95). For the camel, the heterogeneity of blood neutrophils remains an open issue. In addition, the interplay between camel neutrophils and other innate immune cells such as monocyte subsets and macrophages (96) has not yet been studied.

### Camel Monocyte Subsets

Monocytes are circulating immune cells with an essential role in the innate immune defense against pathogens (97). Upon migration into tissues, monocytes are responsible for the replenishment of other immune cells of the mononuclear phagocyte system including macrophages and dendritic cells, which bridge the innate and adaptive immune responses (98, 99). For their effective antimicrobial functions, monocytes are equipped with several receptors enabling pathogen sensing, engulfment, and elimination (100, 101). The cell surface cluster of differentiation (CD) antigens CD172a, CD14, CD16, CD163, and MHCII have been proven as reliable markers to describe monocyte heterogeneity, their functional status, and their polarized differentiation into distinct macrophage subtypes (102–105). CD172a, which is known as signal-regulatory protein alpha (SIRPa), is a glycosylated cell surface receptor expressed on myeloid cells and functions as a regulatory receptor that inhibits cell signaling (106). CD14 is membrane protein mainly expressed on monocytes and functions with TLR-4 as a bacterial pattern recognition receptor responsible for binding lipopolysaccharide (LPS), the cell wall component of gram-negative bacteria (107). CD163 is a scavenger receptor for haptoglobin–hemoglobin complexes that is mainly expressed on monocytes and macrophages and is considered as a marker for anti-inflammatory functional subtypes of these cells (108). Major histocompatibility (MHC) class II molecules are antigen receptors expressed on blood monocytes and B cells and present antigens to T helper cells (109).

**Figure 2:**
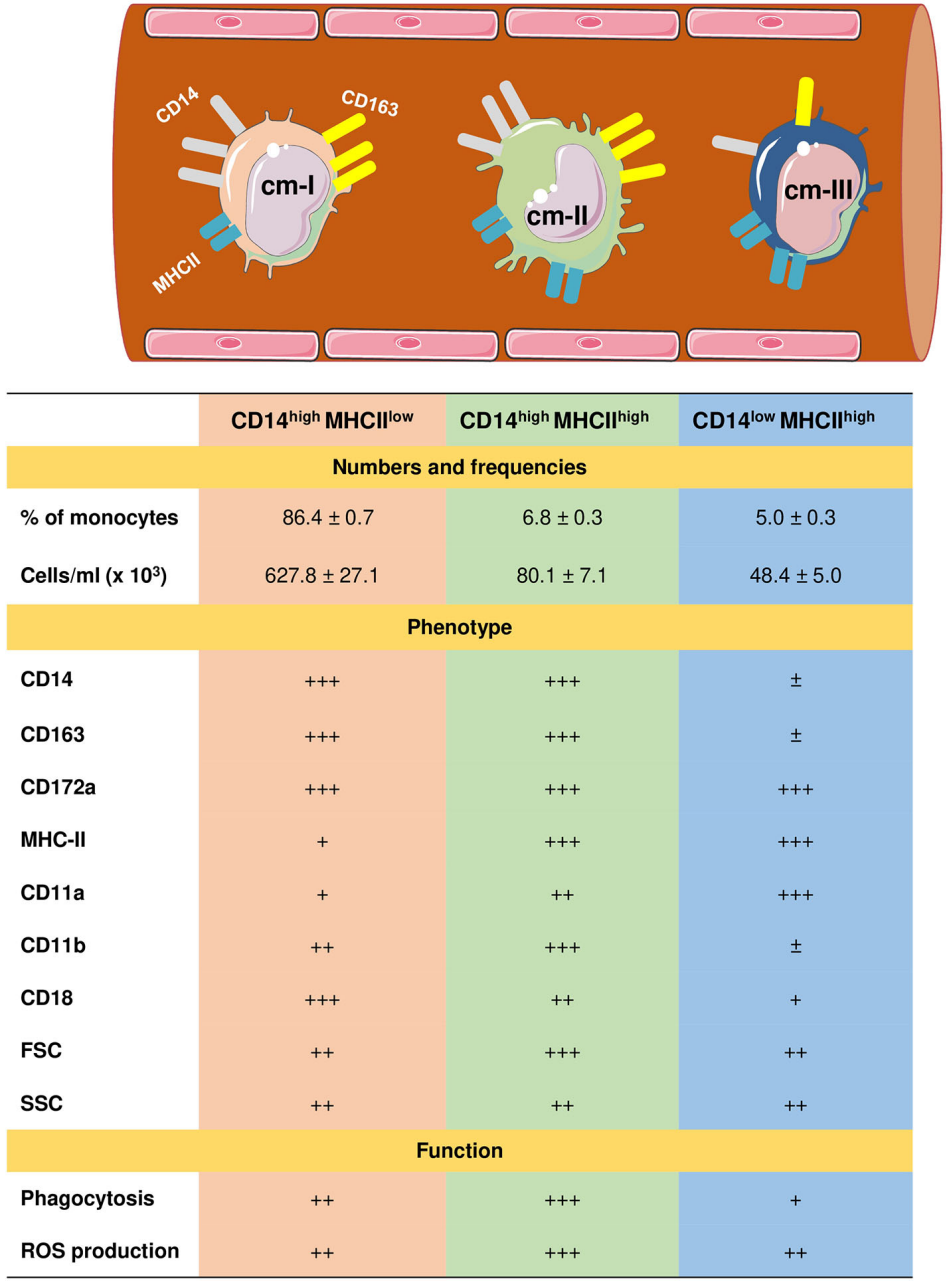
Heterogeneity of camel monocyte subsets. Camel monocytes are subdivided according to the surface expression of CD14 and MHCII into three monocyte subsets. (1) Camel monocyte I (cM-I) with high expression of CD14 and low expression of MHCII (CD14^high^MHCII^low^), cM-II with high levels of both CD14 and MHCII (CD14^high^MHCII^high^) and cM-III with high expression of MHCII but low expression of CD14 (CD14^low^MHCII^high^). The expression levels of cell surface molecules are presented as ± for very low to no-expression, + for weak expression, ++ for intermediate expression, and +++ for high expression. Functional capacities of monocyte subsets are presented as + for weak, ++ for intermediate, and +++ for strong capacity.

Due to the lack of monoclonal antibodies cross-reactive with camel CD16 (59), three subpopulations of monocytes in dromedary camels have recently been identified based on the expression profiles of CD172a, CD14, MHCII, and CD163 (61). Similar to the porcine and bovine systems (104, 106, 110–112), the signal-regulatory protein alpha (CD172a) has been identified as a pan monocyte marker for camel monocytes. The most abundant subset of camel monocytes (87% of total monocytes) expresses high levels of CD14 and CD163, but low levels of MHCII (CD14^high^CD163^high^MHCII^low^) and is classified as camel monocyte (cMo)-I. A small fraction of camel monocytes (6 % of total monocytes) expresses high levels of CD14, CD163, and MHCII (CD14^high^CD163^high^MHCII^high^) and is designated as cMo-II. The third minor monocyte subpopulation cMo-III (5 % of total monocytes) displays high expression of MHCII but low expression of CD14 and CD163 (CD14^low^CD163^low^MHCII^high^) (61) (**Figure 2**).

Different monocyte classification systems have been used in different species (113). Human and bovine monocytes were classified into the major population of CD14++ CD16- classical monocytes and two minor populations of CD14++ CD16+ intermediate monocytes and CD14+ CD16++ non-classical monocytes (104, 106, 112, 114, 115). Due to their low expression of CD14, murine monocytes were classified into three subsets based on their expression of the myeloid markers Ly6C and CD43 (116, 117). Whereas for the analysis of monocyte heterogeneity in the pig (110, 118) and the dog (119), other monocytic markers including CD163, CD172a and MHCII have been used.

The expression of high levels of CD14 and CD163 on cMo-I and the low MHCII expression together with their dominance among all blood monocytes suggests close similarity with bovine and human classical monocytes (104, 115). The phenotypic and functional properties (highest anti-bacterial activity) of cMo-II suggests this subset is an equivalent to human and bovine intermediate monocytes (106, 113). Similarly, high levels of surface MHCII and adhesion molecule leukocyte function-associated antigen (LFA)-1 (α1β2; β2 integrin; CD11a/CD18) and the low expression density of surface CD14 and CD163 together with a reduced phagocytic and ROS generation activity, suggest that cMo-III represent the counterparts of bovine non-classical monocytes (96, 104, 106).

In a recent report, the clinical relevance of camel monocyte subsets in camel clinical endometritis has been investigated (81). In this study, animals with endometritis showed a significant expansion in the fraction of camel inflammatory monocytes (cMo-II). In addition, increased numbers of cMo-II were indicative for the severity of endometritis. The study suggested camel cMo-II as a disease biomarker for clinical endometritis in camels (81).

Camel monocytes appear to be the only leukocyte population that exclusively express CD26, the MersCoV receptor and are therefore suggested to play a key role in either disease pathogenesis or immune response to the virus (86, 120). Whether the mentioned camel monocyte subsets differ in their expression intensity of CD26 is unknown. More work is needed to further explore the subset-specific function in health and disease for camels as for human and bovine monocyte subsets. Their role in the pathogenesis of different infectious and non-infectious diseases has been indicated in a series of recent studies (121–124). A further open question is, whether camel monocytes show subset-specific potential to differentiate into distinct functional subsets of macrophages or dendritic cells.

### Camel Lymphoid Cell Subpopulations

Due to the lack of camel-specific antibodies, only selected subpopulations of camel lymphoid cells are identifiable. Thus, a comparison of camel lymphoid cell populations with cells of other domestic animal species and humans is very limited. Notably, CD8+ T-cells, cytotoxic T cells and NK cells cannot be identified in camels, which severely inhibits the analysis of anti-viral and vaccination responses. Based on the TCR type, T cells are divided into αβ T cells recognizing peptide antigens presented on MHC molecules and γδ T cells recognizing antigen epitopes in an MHC independent manner (125). Using monoclonal antibodies cross-reactive to the camel CD4 antigen, the bovine γδ T cell marker WC1, the B cell antigen GC26A together with monoclonal antibodies specific to CD14 to exclude monocytes, it was possible to identify camel CD4-positive T cells, WC1-positive γδ T cells, and GC26A-positive B cells in the blood of dromedary camels (**Table 2**) (36).

**Table 2:** Phenotypic properties of T cells and B cells in camel blood.

	CD4+ T cells (CD4^+^WC1^-^)	γδ T cells (WC1^+^CD4^-^)	B cells (GC26A^+^MHC-II^+^CD14^-^)
**Frequency in blood**			
% of lymphocytes (Mean ± SEM)	24.6 ± 1.7	7.4 ± 0.3	26.6 ± 1.9
% of lymphocytes (Min. – Max.)	14.2–33.1	1.0–20.1	18.4–42.0
**Phenotype**			
CD4	+++	–	–
WC1	–	+++	–
GC26A	–	–	+++
MHC-II	–	–	+++
CD18	++	++	?
CD11a	++	++	?
CD11b	+	++	?
CD62L	+	++	?
Effector cells	CD11a^high^CD44^high^ (17.0 ± 1.2) %	?	?

The expression levels of cell surface molecules are presented as - for very low to no-expression, + for weak expression, ++ for intermediate expression, and +++ for high expression. Non investigated parameters are presented as ? (36).

In healthy dromedary camels, blood lymphocytes are composed of a major fraction of B cells (mean percentage of 26.6%) followed by CD4-positive T cells (24.6%) and a minor fraction of γδ-T cells (7.4%) (36). In comparison to their nearest relatives (Lamini), healthy camels show some similarties in their lymphocyte composition (126, 127). Similar to their dominance among camel blood lymphocytes, B cells represent the main lymphocyte population in blood from healthy alpacas (126, 127). In addition, the fractions of CD4+ T cells and γδ -T cells in blood from alpacas (126, 127) and dromedary camels (36) are comparable. It is unknown, whether camel CD8+ T cells are present in the same frequency as in blood of alpacas (126, 127). The significant expansion of CD8+ T cells in the gut-associated lymphoid tissue (GALT) of alpacas 9 days postinfection with bovine virus diarrhea virus (BVDV) indicates a key role for this lymphocyte subset in the immune response of camelids to viral infections (127).

The analysis of the expression pattern of the adhesion molecules CD11a, CD11b, CD18, and CD62L, which play essential roles in lymphocyte trafficking to peripheral tissues (128), revealed similar expression patterns on camel and bovine CD4+ T cells and γδ T cells (36, 129).

T helper cells are key players in the adaptive immune response through their essential role in managing both humoral and cell-mediated immune responses. Upon antigen-specific stimulation, naïve CD4+ T cells differentiate into effector T helper cells, which can be distinguished based on the differential expression of cell surface adhesion molecules such as CD45, CD44, CD62L, and CD11a (130–134). Similar to their human counterparts (135), camel naïve (CD11a^low^ CD44^low^) and effector (CD11a^hi^ CD44^hi^) T helper cells have recently been identified with an elevated proportion of effector T helper cells in animals with respiratory infections (23.5% of total CD4-positive lymphocytes compared to 17.1% in healthy camels) (36).

### Camel Cytokines

Functional properties of camel lymphocyte subpopulations have not been investigated so far. Especially the characterization of camel subsets of helper T cells and the innate signals required for their functional polarization into Th1, Th2, or Th17 subsets requires further investigation. T cell polarization is one of the key factors that determine the outcome of infectious diseases (136). The characterization of T effector cell subsets is limited by the lack of monoclonal antibodies specific for camel Th1, Th2, and Th17 cytokines. The characterized genes of Th1 (IL-2, IL-12, and IFN-γ) and Th2 (IL-4, IL-10 and IL-13) cytokines in the Bactrian camel (137, 138), however, could represent a valuable tool for conducting functional studies on T cell polarization in camels. The high homology between Bactrian camels and other species, including the llama, pig, cow, and horse regarding the nucleotide sequences of their cytokine genes (137) also suggests the necessity of testing monoclonal antibodies specific for cytokines of these species for their cross-reactivity with camel cytokines. However, the lack of characterized camel antigen-presenting cells and the establishment of *in vitro* systems for the differentiation of camel monocyte-derived macrophages and monocyte-derived dendritic cells hamper antigen-specific activation and T-cell polarization studies.

Studies on cytokine responses *in vivo* relied on the measurement of mRNA expression. Bactrian camels vaccinated with a live attenuated *Brucella abortus* S19 vaccine responded with an upregulated expression of the Th-1 cytokine IFNγ with low or no expression of the Th2 cytokines IL-10 and IL-4, indicating the activation of a cell-mediated immune response (138). To address the humoral immune response and the production of antigen-specific antibodies, a recent immunization study with ovalbumin proved that the upregulated cytokine expression pattern of Bactrian camel lymphocytes was restricted to Th-2 cytokines (IL-4, IL-10, and IL-13) (139). At present, these studies basically indicate a high degree of similarity in the polarized cytokine response towards vaccines and antigens in other mammalian species, namely cattle (140, 141).

Type 1 interferons represent the most important cytokines in innate immunity during infections with viruses in addition to antitumor immune responses. The camel displays a similar broad spectrum of IFN alpha family members as cattle (142) and humans (143). For instance, eleven IFN-α subtypes (144) and one member of the IFN epsilon family were identified (145). The functional properties of type I interferons appear similar to other mammalian species, including the antiviral effect, the induction of interferon-responsive genes, and the tumor cell cytotoxicity (144, 145). Studies describing other immune-modulatory effects of type I interferons are still lacking (145).

## Camel Immunogenetics

In comparison to other species inhabiting the same geographical area, camels are more resistant to some pathogens (4, 15, 16, 18, 146). The ability to respond to a variety of antigens is affected by the diversity of highly specialized antigen receptors (147). This has been addressed in a series of immunogenomic studies which investigated the polymorphism of genes encoding different camel antigenic receptors, including the αβ and γδ T cell receptor, the NK cell receptor, and the antigen-presenting molecules MHC-class I and class II.

In comparison to other Artiodactyls, dromedary camels display a limited repertoire of T cell receptor delta variable (TRDV) and T cell receptor gamma variable (TRGV) genes (26). However, the diversity of the camel dromedary γδ T cell repertoire is significantly expanded by somatic hypermutation of the TRDV and TRGV genes (27, 28, 148). The diversity of the variable domains of the αβ T cell receptor is formed only by classical combinatorial and junctional diversity and not by somatic hypermutation (148).

Antigen recognition by T cell receptors on CD4+ or CD8+ T cells requires the presentation of antigenic peptides by MHC class II or class I molecules respectively (149, 150). As those polymorphic antigen-presenting molecules display promiscuous and selective interactions with antigen peptides, diversity in genes and alleles encoding for MHC class I and class II molecules contributes directly to the ability of a species to respond towards a range of different pathogens (150). In a recent report, Plasil and coworkers investigated the localization, organization, and sequence of camel MHC genes (23). MHC genes are located on chromosome 20 in camels and are organized in MHC class II, MHC class III, and MHC class I genes, an organization that follows the same pattern as in other mammalian species (148). The camel MHC genomic structure more closely resembles the porcine rather than the bovine MHC. Compared with other mammalian species, however, camels show a significantly lower molecular diversity of both MHC class I and class II genes (23, 24).

Natural killer (NK) cells are innate lymphoid cells with key roles in innate immune responses against intracellular pathogens and tumor cells. These multiple functions are mediated by different activating and inhibitory NK cell surface receptors, which determine the activation status of an individual NK cell (151). A recent work by Futas et al. investigated the diversity of gene families encoding the camel NK cell receptors, including the natural killer complex (NKC) and the leukocyte receptor complex (LRC), which mediate their function through the interaction with MHC class I molecules (25). Collectively, the study identified a low polymorphism of the killer-cell immunoglobulin-like receptors (KLR) genes in camels, which is similar to the polymorphism of this complex in the domestic pig. The study also revealed important differences in the genomic organization and polymorphism of genes encoding NK cell receptors between camels and cattle (25).

Recently, high quality genome assemblies have been developed for domestic and wild camel species (6, 29). Computational methods were employed for the improvement of genome assemblies of the three Old World camel species (6). The authors used the upgraded genome assemblies to investigate nucleotide diversity of immune response genes in the three species. The highest mean nucleotide diversity was identified in the domestic Bactrian camel. The comparison between several innate and adaptive immune response gene groups revealed the highest mean nucleotide diversity in the major histocompatibility complex (6).

The overall reduced antigen receptor diversity and MHC polymorphism, however, indicates the existence of other mechanisms responsible for the higher resistance of camels to infectious diseases. Whether camel-specific epigenetic regulatory mechanisms of adaptive immune responses contribute to the relatively higher resistance to infections is currently unknown.

## The Immune System of the Pregnant She-Camel

Pregnancy is a physiologic condition, usually associated with modulations in different immune mechanisms, which ensure protection against pathogens and at the same time prevent immune mediated destruction of the conceptus (152–154). Immunomodulation during pregnancy is not restricted to the local uterine environment but extends also into the periphery (155, 156). Pregnancy-associated immunomodulation has been addressed by a large number of studies in pregnant women (157), cows (158, 159), mares (160, 161), and sows (162–165). A recent study investigated the impact of pregnancy on the phenotype and function of she-camel blood leukocytes (60). The observed significant leukocytosis of pregnant she-camels is similar to findings reported for pregnant cows (166) and women (167), which is usually linked with an increased cortisol level during pregnancy (161). According to the same study, the leukocytosis in pregnant she-camels is characterized by a reduced neutrophil fraction and higher percentages of lymphocytes and monocytes. This leukocyte composition pattern, however, differs from the pattern reported for pregnant cows (162). In cows, pregnancy is associated with higher fractions of neutrophils, lower fractions of lymphocytes but no changes in the fractions of monocytes (166, 168).

Although not proven, it was suggested that the enhanced neutrophil extravasation and accumulation in the uterine tissue might be responsible for the decreased proportion of neutrophils in the blood, as neutrophils from pregnant animals expressed higher densities of the cell adhesion molecule LFA-1 on their surface than non-pregnant animals (60). Ex vivo functional analyses revealed an enhanced antimicrobial activity of neutrophils from pregnant she-camels. This finding is in line with reports of pregnant mares (169), but is opposite to the dairy cow, where pregnancy is associated with impaired antimicrobial functions of neutrophils (170, 171).

## The Immune System of the Newborn Camel Calf

Similar to horses, pigs, and ruminants, the epitheliochorial placenta of camels does not allow trans-placental passage of maternal immunoglobulins to the fetus (172, 173). Therefore, the newborn camel calf is born without serum immunoglobulins and postnatal protection mainly relies on an adequate absorption of maternal colostral antibodies until the maturation of the calf’s own immune system (174, 175). The transfer of colostral immunoglobulins to the newborn camel calf has been subject of many investigations (176–182). Several immunoglobulin classes, including the IgM, IgG, and IgA, have been identified in the camel colostrum (182). However, only the uptake of maternal IgG, representing the most abundant immunoglobulin in camel colostrum, into the newborn’s blood has been studied.

**Figure 3:**
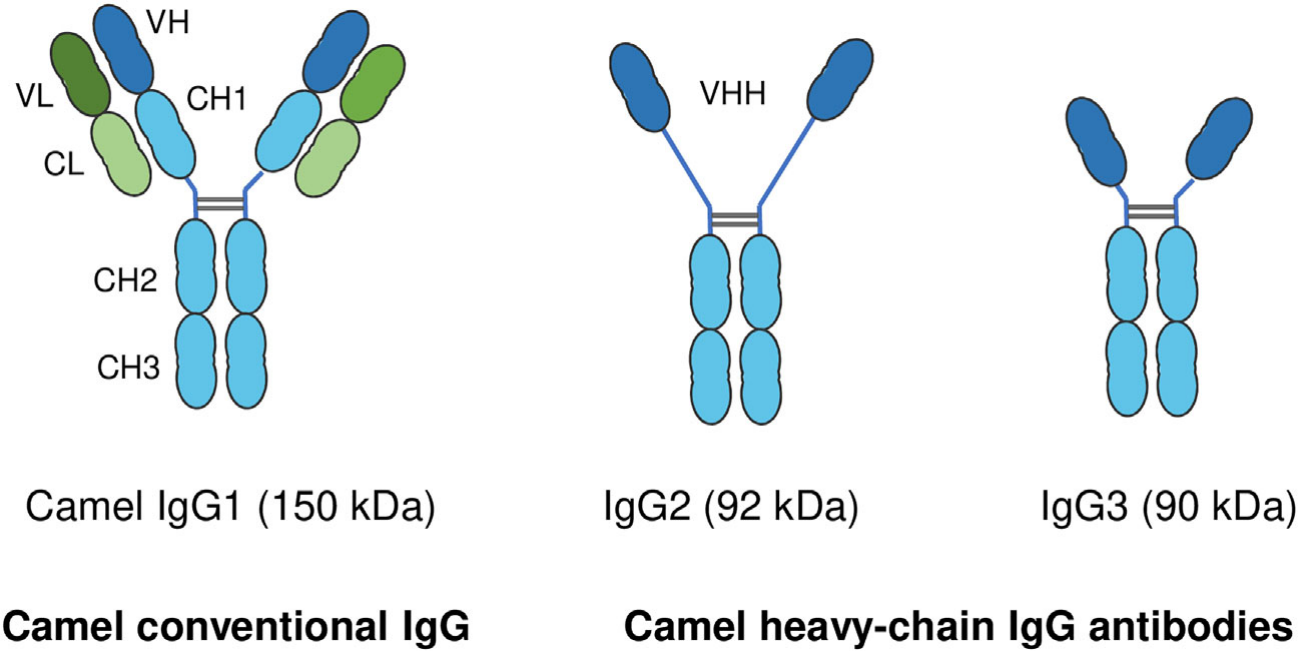
Structure of camel immunoglobulin (Ig) G subclasses. Camel IgGs are currently classified into three structurally different isotypes: Camel IgG1 consists of two identical heavy chains (H) each composed of three constant domains (CH1–CH3) and a single variable domain (VH). Each heavy chain is covalently bound to identical light chains (L) with a constant (CL) and a variable domain (VL). Camel IgG2 and IgG3 are composed of only two identical heavy chains (long-hinge heavy chain in IgG2 and short-hinge heavy chain in IgG3). Camel single-chain IgG subclasses are devoid of the first heavy chain constant region CH1.

In addition to the conventional IgG with its heterodimeric structure, camelids also possess non-conventional single-chain IgG antibodies, which are not found in any other mammalian species (183). In contrast to conventional IgG structure, which consists of two identical heavy chains (H) and two identical light chains (L), camel single-chain IgG antibodies are devoid of the light chain and the first heavy chain constant region CH1. The camel IgG isotype is currently classified into three structurally different subclasses: camel IgG1 with two light and two heavy chains, camel IgG2 with a long-hinge heavy chain, and camel IgG3 with a short-hinge heavy chain (**Figure 3**). The camel heavy-chain antibodies (HCAbs) IgG2 and IgG3, which lack light chains, contribute up to 75% of all serum IgG (13).

Both classical two-chain antibodies (IgG1) and HCAbs (IgG2 and IgG3) are present in camel colostrum (182), and both are involved in the passive transfer of colostral IgG antibodies to the newborn calf (178, 179). Some studies investigated the development of IgG (176) and HCAbs (178) in the blood of the newborn camel calf. The rise in serum IgG levels in calf serum two months after birth is indicative of the production of significant levels of the calf’s own IgG (177, 184). The role of maternal colostral cells in neonatal immune system development, and their responses to vaccination is of growing interest in other species (185–190). However, no studies have yet been conducted on the role of colostral immune cells in the modulation of the camel calf immune system.

Age-related changes of innate and adaptive cellular immune responses have been described for different species (191, 192). The immaturity of newborn immune cells was linked to a higher susceptibility to infectious diseases and higher mortality rates during the early weeks after birth (191, 193–200). As in other ruminants, camel newborns and adult camels differ significantly regarding their leukogram pattern, phenotype and functionality of blood leukocyte subpopulations (201). During their first month of life, the leukogram pattern of newborn camel calves is characterized by higher leukocyte numbers, higher numbers of neutrophils, monocytes, and lymphocytes, but lower numbers of eosinophils in comparison to adult camels (201). The reduced numbers of eosinophils in newborn camel calves, which play a major role in parasitic immunity (39), has been related to lower parasitic manifestation in comparison to adults (202).

High neutrophil to lymphocyte ratio (NLR) has been linked to impaired immune cell function and poor patient survival in different inflammatory diseases (45–47). Camel calves are born with a higher NLR (12.1 in average) than found in adults (5.1 in average) (184). NLRs drop within two months after birth to adult camel values. It was suggested that initially high calf NLRs reflect the pro-inflammatory nature of newborn camel immune responses and a shift towards mature and correctly polarized immune responses takes place in the two-month period after birth (184).

Similar to other artiodactyls such as sheep, cows and pigs, with higher frequencies of blood γδ T cells in younger animals, γδ T cells account for up to 35% of blood lymphocytes in newborn and young camel calves (36, 70). This indicates that camels belong to the γδ-high species, in contrast to γδ-low mammalian species like humans and mice, where γδ T cells represent only a minor subpopulation (< 5%) of circulating lymphocytes (203).

Compared to adult camels, the fraction of B cells among blood lymphoid cells of newborn camels is higher than in adults, whereas the fraction of CD4+ T cells is lower than in adults (200). The authors discussed a link between elevated numbers of circulating leukocyte populations in camels and their lower expression density of cell adhesion molecules (CD11a, CD11b, CD18) compared to adult leukocytes (201).

**Figure 4:**
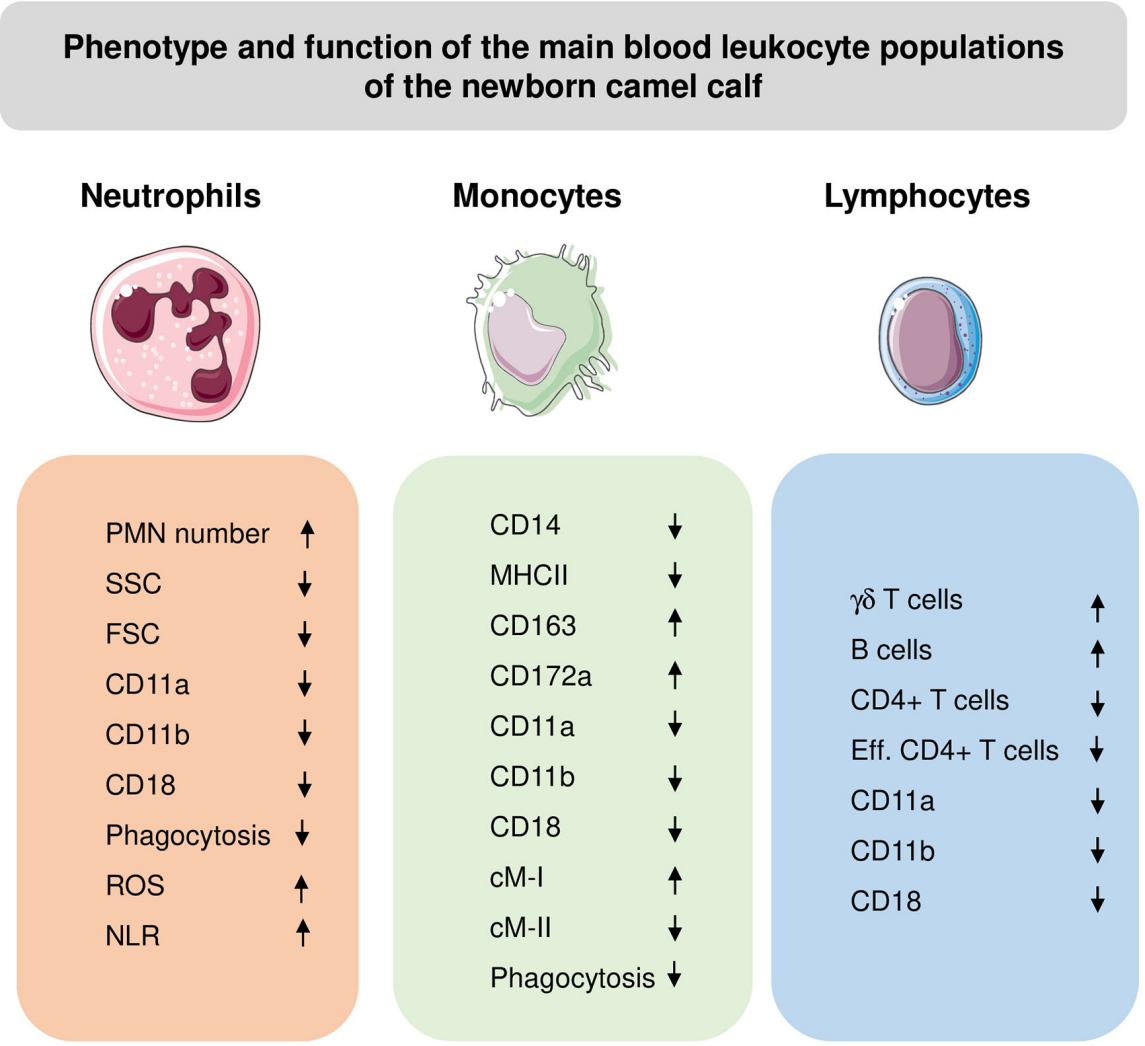
Phenotypic and functional properties of neutrophils (PMN), monocytes, and lymphocytes in blood of newborn camel calves. ROS, Reactive Oxygen Species amount in unstimulated cells; NLR, Neutrophil to lymphocyte ratio; Eff, effector cells. The direction of the arrows indicates higher (up arrow) or lower (down arrow) values for calves compared to adults.

Monocytes from newborn and adult camels showed different expression patterns of the monocyte-related surface molecules, CD172a, CD14, CD163 and MHCII (61) (**Figure 4**). Compared to adult camels, newborns display higher numbers of cMo-I and cMo-III, and less numbers of inflammatory cMo-II (61).

Camel calf leukocytes show functional properties that are different from adult camel leukocytes. Flow cytometric analysis of cell granularity and cell size, which are widely used as indicators of the cell activation status (204, 205), revealed a reduced activation potential of calf leukocytes in comparison to adults (120). Phagocytotic activity of newborn neutrophils and monocytes was found to be lower than in adults, with a lower percentage of phagocytosis-positive cells and a reduced number of bacteria ingested per cell (58).

## Mucosal Immunity in Camels

Mucosal body surfaces are equipped with specialized mucosa-associated lymphoid tissue (MALT), which represent the first defense barrier of the body preventing infectious agents from invading the internal body tissues (206). Therefore, the characterization of mucosal immune mechanisms has essential impact on the understanding of disease pathogenesis of and the development of effective vaccines against mucosal infections, including mastitis, metritis, respiratory infections, and gastrointestinal infections. Several studies adressed anatomical structures of the gastrointestinal MALT of Bactrian and dromedary camels (207–211). Detailed and comparative aspects of immune mechanisms on camel body surfaces are still unkonwn.

The intestine represents the main surface of interaction between the immune system and the huge numbers of microorganisms, which play a pivotal role in guiding the maturation of the mucosal immune system and shaping systemic immunity (212, 213). Morphological studies of the gastrointestinal tract of Bactrian camels revealed a distinct structure and distribution of the MALT in this species (209, 214). Distributed along the whole small intestine, four distinct types of Peyer’s patches, including nodular, faviform, cup-shaped, and cystic form Peyer’s patches, have been identified in Bactrian camels (214). The nodular and cystic forms of Peyer’s patches are unique to this species. The number of Peyer’s patches in the small intestine of Bactrian camels increases with age and peaks in 5-year-old camels followed by a subsequent decline (214). Peyer’s patches in the large intestine of Bactrian camels are mainly located on the surface of the entire ileocecal orifice, the beginning of the cecum, and the first third of the colon. The ileocecal orifice has been suggested as the main inductive site for mucosal immune responses in the Bactrian camel large intestine (209). In the dromedary camel, Peyer’s patches have cup-shaped structures and are distributed in the anti-mesenteric side of the ileum (211). They are not present in the jejunum or duodenum (215). Whether the distinct morphology, structure, and distribution of the MALT structures in camels are reflected by species-specific functional differences of mucosal immune responses, is currently unknown. Mucosal immunoglobulins contribute to the immune homeostasis at the mucosal interface (216). The distribution of secretory IgA (SIgA) and IgG-secreting cells (ISCs) in the lamina propria of the small intestine of Bactrian camels suggests their significant contribution to mucosal immunity in this species (210, 217). Similar to the age-related changes in the number of PP in the intestine of Bactrian camels, SIgA and IgG ISC numbers increase with age with a peak at puberty (210, 217).

### The Camel Immune Response to Middle East Respiratory Syndrome Coronavirus

Middle East respiratory syndrome Coronavirus (MERS-CoV) is an emerging zoonotic pathogen that causes the Middle East Respiratory Syndrome (MERS) (11, 218–220). Dromedary camels are considered the only confirmed animal host for MERS-CoV and the source of zoonotic infection (221–228). In humans, MERS-CoV infection is associated with either hospitalization or death, while MERS-CoV-infected camels show only mild and transient respiratory symptoms with no need for veterinary care (15). It is unknown whether special host-pathogen interaction mechanisms in camels contribute to the higher resistance of this species to MERS.

The very high seroprevalence rates (74–100%) of MERS-CoV in camel populations in Africa and the Arabian Peninsula indicate high infection and transmission rates of the virus in camels (16). The dipeptidyl peptidase 4 (DPP4; CD26), a type II transmembrane glycoprotein involved in cleavage of dipeptides and degradation of incretins (229), has been identified as a functional receptor for the MERS-CoV (230–232). The differential expression of DPP4 in the respiratory tracts of humans and camels has been suggested as the primary cause of limited MERS-CoV replication in the human upper respiratory tract and hence restrict transmission between humans. While DPP4 is only expressed in the human lower respiratory tract epithelium, only the upper respiratory tract epithelium of camels show DPP4 expression (233). Whether the interaction of MERS-CoV with epithelial cells in the lower (human) or upper respiratory tract (camel) results in the secretion of location-specific mediators, which may differently modulate the onset and resolution of subsequent innate and adaptive immune mechanisms, is still an open question.

In opposite to humans, where DPP4 is mainly found on human T lymphocytes rather than monocytes (234), dromedary camels display the highest expression of DPP4 on blood monocytes (86, 120). This may indicate different roles for innate and adaptive immune responses to MERS-CoV in the two species. This is also supported by recent observations on MERS-CoV-infected human individuals, where gradual increases in blood lymphocyte count during MERS progression was observed in all the survivors, whereas the response in diseased patients was characterized by lymphopenia and increased neutrophils and monocytes counts (235).

One of the potential control strategies of MERS-CoV relies on reducing virus transmission from animals to humans through vaccination of camels (223, 236, 237). The development of protective MERS-CoV vaccines for dromedary camels, however, requires an in depth understanding of local immune mechanisms in the respiratory tract in camels and the identification of correlates of protection against the virus. In MERS patients, the development of neutralizing antibodies was not sufficient for an effective clearance of the virus (238). The association between the recovery from MERS and the generation of both antibody and T-cell responses (221, 239) indicates key roles for cell-mediated immune mechanisms against the virus. However, the analysis of immune responses of camels to MERS-CoV infection was limited to the investigation of virus-specific antibodies (179). Studies on cell-mediated immune responses are still lacking. The characterization of camel NK and cytotoxic T cells and their role in anti-viral immunity in the context of infection with MERS-CoV is one of the promising lines of research. MERS-CoV naturally infected camels are currently discussed as a challenge model in vaccine efficacy studies (16). The characterization of mucosal immune mechanisms in the camel respiratory tract, including detailed phenotypic and functional analyses of immune cells in bronchoalveolar lavages and lung parenchymas of MERS-CoV-infected and recovered camels would be a prerequisite for the elucidation of MERS-CoV pathogenesis in these animals.

## Conclusions

The camel represents a multipurpose domestic animal used for meat and milk production, racing, and transportation. Different components of the cellular immune system of the dromedary camel show several species-specific phenotypic and functional properties. In contrast to other domestic species, the camel leukogram is dominated by the neutrophil fraction resulting in a higher neutrophil to lymphocyte ratio. Camel monocytes are classified into three phenotypically and functionally different subsets based on the expression of the surface molecules CD14 and MHCII with many similarities with the bovine monocyte classification system. Camels belong to the γδ T cell-high species with especially high percentages of γδ T cells in newborn and young animals. Circulating camel newborn immune cells contain lower numbers of inflammatory monocytes, show a reduced expression of cell adhesion molecules on all leukocytes, and a reduced antimicrobial functionality of monocytes and neutrophils.

Despite the progress achieved in the field of camel immunology, there are still many gaps towards a more profound understanding of the camel immune system. Open questions cover the innate recognition mechanisms, the functional characterization of macrophages and dendritic cells, and their signals responsible for T cell activation and polarization toward distinct functional subtypes such as type-1, type-2, or type-17 cells. The characterization of camel NK and cytotoxic T cells and their role in anti-viral immunity, especially in the context of infection with the zoonotic pathogen MERS-CoV is still in its infancy. Promising lines of research would also include host-pathogen interactions on camel mucosal body surfaces such as the respiratory tract, the mammary gland, the uterus, and the intestine.

A deeper characterization of camel infection immunity would help to identify protection-relevant immune mechanisms, essential for the design of effective vaccines, the identification of disease biomarkers, and the selection of animals with higher disease resistance.

## Author Contributions

JH and HS wrote the manuscript. All authors contributed to the article and approved the submitted version.

## Conflict of Interest

The authors declare that the research was conducted in the absence of any commercial or financial relationships that could be construed as a potential conflict of interest.

## References

[1] Jirimutu Wang Z Ding G Chen G Sun Y Sun Z Genome sequences of wild and domestic bactrian camels. Nat Commun (2012) 3:1202. 10.1038/ncomms2192 23149746 PMC3514880

[2] Andersen HT [Desert, man and camel]. Nord Med (1966) 75:61–3. 5903328

[3] Yousif OK Babiker SA The desert camel as a meat animal. Meat Sci (1989) 26:245–54. 10.1016/0309-1740(89)90010-7 22055021

[4] Burger PA The history of Old World camelids in the light of molecular genetics. Trop Anim Health Prod (2016) 48:905–13. 10.1007/s11250-016-1032-7 PMC4884201 27048619

[5] Ali A Baby B Vijayan R From Desert to Medicine: A Review of Camel Genomics and Therapeutic Products. Front Genet (2019) 10:17. 10.3389/fgene.2019.00017 30838017 PMC6389616

[6] Lado S Elbers JP Rogers MF Melo-Ferreira J Yadamsuren A Corander J Nucleotide diversity of functionally different groups of immune response genes in Old World camels based on newly annotated and reference-guided assemblies. BMC Genomics (2020) 21:606. 10.1186/s12864-020-06990-4 32883205 PMC7468183

[7] Dorman AE The camel in health and disease. 2. Aspects of the husbandry and management of the genus Camelus. Br Vet J (1984) 140:616–33. 10.1016/0007-1935(84)90013-7 6391603

[8] Kebede F Gelaty E Studies on major respiratory diseases of Camel (Camelus dromedarius) in Northeastern Ethiopia. Afr J Microbiol Res (2010) 4:1560–4. 10.5897/AJMR.9000669

[9] Al-Ruwaili MA Khalil OM Selim SA Viral and bacterial infections associated with camel (Camelus dromedarius) calf diarrhea in North Province, Saudi Arabia. Saudi J Biol Sci (2012) 19:35–41. 10.1016/j.sjbs.2011.10.001 23961160 PMC3730540

[10] Al-Ani FK Domestication, distribution and population. In: Al-Ani F, editor. Camel Management and Diseases. Jordan: Al-Sharq printing press (2004). p. 1–24.

[11] Drosten C Meyer B Muller MA Corman VM Al-Masri M Hossain R Transmission of MERS-coronavirus in household contacts. N Engl J Med (2014) 371:828–35. 10.1056/NEJMoa1405858 25162889

[12] Muyldermans S Nanobodies: natural single-domain antibodies. Annu Rev Biochem (2013) 82:775–97. 10.1146/annurev-biochem-063011-092449 23495938

[13] Arbabi-Ghahroudi M Camelid Single-Domain Antibodies: Historical Perspective and Future Outlook. Front Immunol (2017) 8:1589. 10.3389/fimmu.2017.01589 29209322 PMC5701970

[14] Jovcevska I Muyldermans S The Therapeutic Potential of Nanobodies. BioDrugs (2020) 34:11–26. 10.1007/s40259-019-00392-z 31686399 PMC6985073

[15] Al Sulayyim HJ Khorshid SM Al Moummar SH Demographic, clinical, and outcomes of confirmed cases of Middle East Respiratory Syndrome coronavirus (MERS-CoV) in Najran, Kingdom of Saudi Arabia (KSA); A retrospective record based study. J Infect Public Health (2020) 13:1342–6. 10.1016/j.jiph.2020.04.007 PMC7180355 32354534

[16] Alharbi NK Ibrahim OH Alhafufi A Kasem S Aldowerij A Albrahim R Challenge infection model for MERS-CoV based on naturally infected camels. Virol J (2020) 17:77. 10.1186/s12985-020-01347-5 32552831 PMC7298446

[17] Bitter H Disease resistance in dromedaries with particular reference to Trypanosoma evansi infection. Hanover, Germany: Tierartliche Hochschule (1986).

[18] Larska M Wernery U Kinne J Schuster R Alexandersen G Alexandersen S Differences in the susceptibility of dromedary and Bactrian camels to foot-and-mouth disease virus. Epidemiol Infect (2009) 137:549–54. 10.1017/S0950268808001088 18687160

[19] Hoter A Rizk S Naim HY Cellular and Molecular Adaptation of Arabian Camel to Heat Stress. Front Genet (2019) 10:588. 10.3389/fgene.2019.00588 31275361 PMC6593249

[20] Ruiz-Saenz J Goez Y Tabares W Lopez-Herrera A Cellular receptors for foot and mouth disease virus. Intervirology (2009) 52:201–12. 10.1159/000226121 19556802

[21] Ulmasov HA Karaev KK Lyashko VN Evgen’ev MB Heat-shock response in camel (Camelus dromedarius) blood cells and adaptation to hyperthermia. Comp Biochem Physiol B (1993) 106:867–72. 10.1016/0305-0491(93)90043-5 8299349

[22] Majeed EN Effects of heat on camel platelet structure and function–a comparative study with humans. Platelets (2009) 20:528. 10.3109/09537100903207513 19852694

[23] Plasil M Mohandesan E Fitak RR Musilova P Kubickova S Burger PA The major histocompatibility complex in Old World camelids and low polymorphism of its class II genes. BMC Genomics (2016) 17:167. 10.1186/s12864-016-2500-1 26931144 PMC4774177

[24] Plasil M Wijkmark S Elbers JP Oppelt J Burger PA Horin P The major histocompatibility complex of Old World camelids: Class I and class I-related genes. HLA (2019) 93:203–15. 10.1111/tan.13510 30828986

[25] Futas J Oppelt J Jelinek A Elbers JP Wijacki J Knoll A Natural Killer Cell Receptor Genes in Camels: Another Mammalian Model. Front Genet (2019) 10:620. 10.3389/fgene.2019.00620 31312212 PMC6614441

[26] Ciccarese S Vaccarelli G Lefranc MP Tasco G Consiglio A Casadio R Characteristics of the somatic hypermutation in the Camelus dromedarius T cell receptor gamma (TRG) and delta (TRD) variable domains. Dev Comp Immunol (2014) 46:300–13. 10.1016/j.dci.2014.05.001 24836674

[27] Antonacci R Mineccia M Lefranc MP Ashmaoui HM Lanave C Piccinni B Expression and genomic analyses of Camelus dromedarius T cell receptor delta (TRD) genes reveal a variable domain repertoire enlargement due to CDR3 diversification and somatic mutation. Mol Immunol (2011) 48:1384–96. 10.1016/j.molimm.2011.03.011 21511341

[28] Vaccarelli G Antonacci R Tasco G Yang F Giordano L El Ashmaoui HM Generation of diversity by somatic mutation in the Camelus dromedarius T-cell receptor gamma variable domains. Eur J Immunol (2012) 42:3416–28. 10.1002/eji.201142176 22961631

[29] Ming L Wang Z Yi L Batmunkh M Liu T Siren D Chromosome-level assembly of wild Bactrian camel genome reveals organization of immune gene loci. Mol Ecol Resour (2020) 20:770–80. 10.1111/1755-0998.13141 32012460

[30] Al-Mujalli A Al-Naeem A Al-Ghamdi G A-SA Al-Yamani E Shehata AM Al-Dubaib M Cellular and biochemical blood profile in camels suffering from dubduba syndrome. Sci J King Faisal Univ (2011) 12:165–72.

[31] Al-Busadah K Some biochemical and haematological indices in different breeds of camel in Saudi Arabia. Sci J King Faisal Univ (2007) 8:131–42.

[32] Zongping L Studies on the haematology and trace element status of adult Bactrian camels (Camelus bactrianus) in China. Vet Res Commun (2003) 27:397–405. 10.1023/a:1024762205249 14509454

[33] Higgins AJ The camel in health and disease. Introduction. Br Vet J (1984) 140:482–4. 10.1016/0007-1935(84)90044-7 6487998

[34] De Genst E Saerens D Muyldermans S Conrath K Antibody repertoire development in camelids. Dev Comp Immunol (2006) 30:187–98. 10.1016/j.dci.2005.06.010 16051357

[35] Hussen J Flow cytometric analysis of phenotype and composition of peripheral blood leukocytes in young and old dromedary camels (Camelus dromedarius). J Camel Pract And Res (2018) 25:1–8. 10.5958/2277-8934.2018.00004.8

[36] Hussen J Shawaf T Al-herz AI Alturaifi HR Al khamees M Alluwaimi AM Expression Patterns of Cell Adhesion Molecules on CD4+ T Cells and WC1+ T Cells in the Peripheral Blood of Dromedary Camels. Pakistan Vet J (2018) 38:231–6. 10.29261/pakvetj/2018.055

[37] Ali A Tharwat M Al-Sobayil FA Hormonal, biochemical, and hematological profiles in female camels (Camelus dromedarius) affected with reproductive disorders. Anim Reprod Sci (2010) 118:372–6. 10.1016/j.anireprosci.2009.08.014 19815355

[38] Vap L Bohn AA Hematology of camelids. Vet Clin North Am Exot Anim Pract (2015) 18:41–9. 10.1016/j.cvex.2014.09.010 25421025

[39] Roland L Drillich M Iwersen M Hematology as a diagnostic tool in bovine medicine. J Vet Diagn Invest (2014) 26:592–8. 10.1177/1040638714546490 25121728

[40] Jones ML Allison RW Evaluation of the ruminant complete blood cell count. Vet Clin North Am Food Anim Pract (2007) 23:377–402, v. 10.1016/j.cvfa.2007.07.002 17920454

[41] Icel E Ucak T Karakurt Y Yilmaz H Tasli NG Turk A The Relation of Neutrophil to Lymphocyte Ratio and Platelet to Lymphocyte Ratio with High Axial Myopia. Ocul Immunol Inflammation (2019) 28:396–40. 10.1080/09273948.2019.1588334 30994377

[42] Alan S Tuna S Turkoglu EB The relation of neutrophil-to-lymphocyte ratio, platelet-to-lymphocyte ratio, and mean platelet volume with the presence and severity of Behcet’s syndrome. Kaohsiung J Med Sci (2015) 31:626–31. 10.1016/j.kjms.2015.10.010 PMC11916011 26709224

[43] Sari I Sunbul M Mammadov C Durmus E Bozbay M Kivrak T Relation of neutrophil-to-lymphocyte and platelet-to-lymphocyte ratio with coronary artery disease severity in patients undergoing coronary angiography. Kardiol Pol (2015) 73:1310–6. 10.5603/KP.a2015.0098 25987404

[44] Wang J Kalhor N Hu J Wang B Chu H Zhang B Pretreatment Neutrophil to Lymphocyte Ratio Is Associated with Poor Survival in Patients with Stage I-III Non-Small Cell Lung Cancer. PloS One (2016) 11:e0163397. 10.1371/journal.pone.0163397 27695079 PMC5047446

[45] Faria SS Fernandes PC Jr. Silva MJ Lima VC Fontes W Freitas-Junior R The neutrophil-to-lymphocyte ratio: a narrative review. Ecancermedicalscience (2016) 10:702. 10.3332/ecancer.2016.702 28105073 PMC5221645

[46] Kaushik R Gupta M Sharma M Jash D Jain N Sinha N Diagnostic and Prognostic Role of Neutrophil-to-Lymphocyte Ratio in Early and Late Phase of Sepsis. Indian J Crit Care Med (2018) 22:660–3. 10.4103/ijccm.IJCCM_59_18 PMC6161585 30294133

[47] Hasselbalch IC Sondergaard HB Koch-Henriksen N Olsson A Ullum H Sellebjerg F The neutrophil-to-lymphocyte ratio is associated with multiple sclerosis. Mult Scler J Exp Transl Clin (2018) 4:2055217318813183. 10.1177/2055217318813183 30515298 PMC6262498

[48] Azari O Molaei MM Emadi L Sakhaee E Sharifi H Mehdizadeh S Haematological and biochemical alterations caused by epidural and intramuscular administration of xylazine hydrochloride in dromedary camels (Camelus dromedarius). Vet Ital (2012) 48:313–21. 23038078

[49] Chaudhary ZI Iqbal J Incidence, biochemical and haematological alterations induced by natural trypanosomosis in racing dromedary camels. Acta Trop (2000) 77:209–13. 10.1016/S0001-706X(00)00142-X 11080512

[50] Mohamed HA Hussein AN Studies on normal haematological and serum biochemical values of the ‘Hijin’ racing camels (Camelus dromedarius) in Kuwait. Vet Res Commun (1999) 23:241–8. 10.1023/a:1006253210461 10461801

[51] Haroun EM Mahmoud OM Magzoub M Abdel Hamid Y Omer OH The haematological and biochemical effects of the gastrointestinal nematodes prevalent in camels (Camelus dromedarius) in central Saudi Arabia. Vet Res Commun (1996) 20:255–64. 10.1007/BF00366923 8739524

[52] Ali BH el Sanhouri AA Musa BE Some clinical, haematological and biochemical effects of four tranquilizers in camels (Camelus dromedarius). Rev Elev Med Vet Pays Trop (1989) 42:13–7. 2740562

[53] Barakat M Abdel-Fattah M Biochemical analysis of normal camel blood. Zbl Vet Med A (1970) 17:550–7. 10.1111/j.1439-0442.1970.tb00808.x 4988387

[54] Soliman MK Shaker M Cytological and biochemical studies on the blood of adult she camels. Indian Vet J (1967) 44:989–95. 5587781

[55] MacHugh ND Bensaid A Howard CJ Davis WC Morrison WI Analysis of the reactivity of anti-bovine CD8 monoclonal antibodies with cloned T cell lines and mouse L-cells transfected with bovine CD8. Vet Immunol Immunopathol (1991) 27:169–72. 10.1016/0165-2427(91)90096-U 1902341

[56] Naessens J Olubayo RO Davis WC Hopkins J Cross-reactivity of workshop antibodies with cells from domestic and wild ruminants. Vet Immunol Immunopathol (1993) 39:283–90. 10.1016/0165-2427(93)90190-F 8310653

[57] Maecker HT McCoy JP Nussenblatt R Standardizing immunophenotyping for the Human Immunology Project. Nat Rev Immunol (2012) 12:191–200. 10.1038/nri3158 22343568 PMC3409649

[58] Hussen J Antibacterial functions of neutrophil and monocyte in newborn dromedary camel calves. J Camel Pract And Res (2019) 26:251–4. 10.5958/2277-8934.2019.00039.0

[59] Hussen J Shawaf T Al-herz AI Alturaifi HR Alluwaimi AM Reactivity of commercially available monoclonal antibodies to human CD antigens with peripheral blood leucocytes of dromedary camels (Camelus dromedarius. Open Vet J (2017) 7:150–3. 10.4314/ovj.v7i2.12 PMC5471749 28652982

[60] Hussen J Shawaf T Al-Mubarak AIA Al Humam NA Almathen F Schuberth HJ Leukocytes immunophenotype and phagocytosis activity in pregnant and nonpregnant dromedary she camels. Pak Vet J (2019) 40:239–43. 10.29261/pakvetj/2019.117

[61] Hussen J Shawaf T Al-Mubarak AIA Al Humam NA Almathen F Schuberth HJ Dromedary camel CD14(high) MHCII(high) monocytes display inflammatory properties and are reduced in newborn camel calves. BMC Vet Res (2020) 16:62. 10.1186/s12917-020-02285-8 32070351 PMC7027094

[62] Hussen J Shawaf T,MJ Schuberth HJ Whole blood stimulation with lipopolysaccharide modulates phenotype and function of dromedary camel neutrophils. J Camel Pract And Res (2019) 26:105–10. 10.5958/2277-8934.2019.00015.8

[63] Lamo D Gahlawat G Kumar S Bharti VK Ranjan P Kumar D Morphometric, haematological and physio-biochemical characterization of Bactrian (Camelus bactrianus) camel at high altitude. BMC Vet Res (2020) 16:291. 10.1186/s12917-020-02481-6 32795315 PMC7427938

[64] Amin AS Abdoun KA Abdelatif AM Seasonal variation in blood constituents of one-humped camel (Camelus dromedarius). Pak J Biol Sci (2007) 10:1250–6. 10.3923/pjbs.2007.1250.1256 19069924

[65] Snow DH Billah A Ridha A Effects of maximal exercise on the blood composition of the racing camel. Vet Rec (1988) 123:311–2. 10.1136/vr.123.12.311 3195011

[66] El-Malky OM Mostafa TH Abd El-Salaam AM Ayyat MS Effect of reproductive disorders on productivity and reproductive efficiency of dromedary she-camels in relation to cytokine concentration. Trop Anim Health Prod (2018) 50:1079–87. 10.1007/s11250-018-1533-7 29423707

[67] Youssef SY Yasien S Mousa WM Nasr SM El-Kelesh EA Mahran KM Vector identification and clinical, hematological, biochemical, and parasitological characteristics of camel (Camelus dromedarius) theileriosis in Egypt. Trop Anim Health Prod (2015) 47:649–56. 10.1007/s11250-015-0771-1 25677167

[68] Petrelli F Dahir A Mohamed A Moretti P Blood values in clinically normal African camels (Camelus dromedarius) of various age. Boll Scient Fac Zootech Vet Univ Naz Somalia (1982) 3:133–7.

[69] Nassar S Mansour S Lofti L Influence of sex on the normal blood picture of adult Egyptian camel (Camelus dromedarius). Assiut Vet Med J (1977) 4:43–50.

[70] Majeed M Hur G Rahman Z Ahmad A Effect of sex and season on 10 haematological values of normal adult one-humped camel. Rev Elev Méd Vét Pays Trop (1980) 37:313–7. 10.19182/REMVT.8222 7455286

[71] Ibrahim A Abdel GA Gameel A Nayel N Abdel Gaffar A El Galaini M A note on the haemogram of the dromedary camel in Bahrain. Rev Elev Méd Vét Pays Trop (1992) 45:318–20. 10.19182/REMVT.8925

[72] Mohammed A Sackey A Tekdek L Gefu J Mean haematological characteristics of healthy adult one humped camel (Camelus dromedarius) introduced into a sub-humid climate in Nigeria. J Camel Pract Res (2008) 15:187–90.

[73] Babeker E Elmansoury Y Suleem A The influence of seasons on blood constituents of dromedary camel (Camelus dromedarius). Online J Anim Feed Res (2011) 3:1–8.

[74] Mehrotra V Gupta M Seasonal variations in certain blood constituents in camel. Indian J Anim Sci (1989) 59:1559–61.

[75] Njiru Z Olaho-Mukani W Khaemba B Ochieng R Ndung’u J Haematological and serological changes during acute Trypanosoma evansi infection in dromedary camel (Camelus dromedarius). J Camel Pract Res (2000) 7:113–6.

[76] Hussein M Hassan H Bilal H Basmae’il S Younis T Al Motlaq A Cephalopina titillator (Clark 1797) infection in Saudi Arabian camels. Zbl Vet Med B (1983) 30:553–8. 10.1111/j.1439-0450.1983.tb01882.x 6650025

[77] Tibary A Fite C Anouassi A Sghiri A Infectious causes of reproductive loss in camelids. Theriogenology (2006) 66:633–47. 10.1016/j.theriogenology.2006.04.008 PMC7103124 16697037

[78] Tibary A Rodriguez J Sandoval S Reproductive emergencies in camelids. Theriogenology (2008) 70:515–34. 10.1016/j.theriogenology.2008.04.024 PMC7103122 18514807

[79] Tibary A Reproductive physiology in the female camelidae in: A. Tibary, (Ed.), Theriogenology in camelidae: anatomy, physiology BSE, pathology and artificial breeding. Rabat, Morocco: Institut Agronomique et Veterinaire Hassen (1997). p. 317–68.

[80] Wernerry UK BN Reproductive disorders in dromedary camels due to infectious causes and its treatment. J Camel Pract Res (1994) 1:85–7.

[81] Hussen J Shawaf T Al-Mubarak AIA Al Humam NA Almathen F Schuberth HJ Leukocyte populations in peripheral blood of dromedary camels with clinical endometritis. Anim Reprod Sci (2020) 222:106602. 10.1016/j.anireprosci.2020.106602 32980651

[82] Refaat D Ali A Saeed EM Al-Sobayil F Al-Samri A Elbehiry A Diagnostic evaluation of subclinical endometritis in dromedary camels. Anim Reprod Sci (2020) 215:106327. 10.1016/j.anireprosci.2020.106327 32216929

[83] Ungar-Waron H Yagil R Brenner J Paz R Partosh N Van Creveld C Reactions of peripheral blood mononuclear cells (PBMC) of camels with monoclonal antibodies against ruminant leukocytes. Comp Immunol Microbiol Infect Dis (2003) 26:137–43. 10.1016/S0147-9571(02)00037-1 12493494

[84] Zidan M Schuberth HJ Pabst R Immunohistology of the splenic compartments of the one humped camel (Camelus dromedarius). Vet Immunol Immunopathol (2000) 74:17–29. 10.1016/S0165-2427(00)00153-7 10760387

[85] Mosaad AA Elbagory AR Khalid AM Waters W Tibary A Hamilton MJ Identification of monoclonal antibody reagents for use in the study of the immune response to infectious agents in camel and water buffalo. J Camel Pract Res (2006) 13:91–101.

[86] Al-Mubarak AIA Differential expression of the coronavirus (Mers-cov) Receptor, Dipeptidyl Peptidase 4, on normal and stimulated leukocytes of dromedary camels. J Camel Pract Res (2018) 25:249. 10.5958/2277-8934.2018.00033.4

[87] Al-Mohammed Salem K Badi FA Al Haroon AI Alluwaimi AM The Cellular Populations of Normal Camel (Camelus dromedaries) Milk. Open J Vet Med (2012b) 2:262. 10.4236/ojvm.2012.24042

[88] Zidan M Kassem A Pabst R Megakaryocytes and platelets in the spleen of the dromedary camel (Camelus dromedarius). Anat Histol Embryol (2000) 29:221–4. 10.1046/j.1439-0264.2000.00268.x 11008369

[89] Al-Ashqar RA Al-Mohammad Salem KM Al Herz AK Al-Haroon AI Alluwaimi AM The CD markers of camel (Camelus dromedarius) milk cells during mastitis: the LPAM-1 expression is an indication of possible mucosal nature of the cellular trafficking. Res Vet Sci (2015) 99:77–81. 10.1016/j.rvsc.2015.01.011 25666226

[90] Kolaczkowska E Kubes P Neutrophil recruitment and function in health and inflammation. Nat Rev Immunol (2013) 13:159–75. 10.1038/nri3399 23435331

[91] Sohn EJ Paape MJ Bannerman DD Connor EE Fetterer RH Peters RR Shedding of sCD14 by bovine neutrophils following activation with bacterial lipopolysaccharide results in down-regulation of IL-8. Vet Res (2007) 38:95–108. 10.1051/vetres:2006052 17156740

[92] Leliefeld PHC Pillay J Vrisekoop N Heeres M Tak T Kox M Differential antibacterial control by neutrophil subsets. Blood Adv (2018) 2:1344–55. 10.1182/bloodadvances.2017015578 PMC5998927 29895625

[93] Rosales C Neutrophil: A Cell with Many Roles in Inflammation or Several Cell Types? Front Physiol (2018) 9:113. 10.3389/fphys.2018.00113 29515456 PMC5826082

[94] Silvestre-Roig C Fridlender ZG Glogauer M Scapini P Neutrophil Diversity in Health and Disease. Trends Immunol (2019) 40:565–83. 10.1016/j.it.2019.04.012 PMC7185435 31160207

[95] Silvestre-Roig C Hidalgo A Soehnlein O Neutrophil heterogeneity: implications for homeostasis and pathogenesis. Blood (2016) 127:2173–81. 10.1182/blood-2016-01-688887 27002116

[96] Hussen J Koy M Petzl W Schuberth HJ Neutrophil degranulation differentially modulates phenotype and function of bovine monocyte subsets. Innate Immun (2016) 22:124–37. 10.1177/1753425915620911 26644394

[97] Jakubzick CV Randolph GJ Henson PM Monocyte differentiation and antigen-presenting functions. Nat Rev Immunol (2017) 17:349–62. 10.1038/nri.2017.28 28436425

[98] Faivre V Lukaszewicz AC Alves A Charron D Payen D Haziot A Human monocytes differentiate into dendritic cells subsets that induce anergic and regulatory T cells in sepsis. PloS One (2012) 7:e47209. 10.1371/journal.pone.0047209 23071758 PMC3468528

[99] Italiani P Boraschi D From Monocytes to M1/M2 Macrophages: Phenotypical vs. Functional Differentiation. Front Immunol (2014) 5:514. 10.3389/fimmu.2014.00514 25368618 PMC4201108

[100] Auffray C Fogg D Garfa M Elain G Join-Lambert O Kayal S Monitoring of blood vessels and tissues by a population of monocytes with patrolling behavior. Science (2007) 317:666–70. 10.1126/science.1142883 17673663

[101] Ziegler-Heitbrock HW Definition of human blood monocytes. J Leukoc Biol (2000) 67:603–6. 10.1002/jlb.67.5.603 10810998

[102] Thawer SG Mawhinney L Chadwick K de Chickera SN Weaver LC Brown A Temporal changes in monocyte and macrophage subsets and microglial macrophages following spinal cord injury in the Lys-Egfp-ki mouse model. J Neuroimmunol (2013) 261:7–20. 10.1016/j.jneuroim.2013.04.008 23711349

[103] Schwartz Y Svistelnik AV Functional phenotypes of macrophages and the M1-M2 polarization concept. Part I. Proinflammatory phenotype. Biochem (Mosc) (2012) 77:246–60. 10.1134/S0006297912030030 22803942

[104] Hussen J Schuberth HJ Heterogeneity of Bovine Peripheral Blood Monocytes. Front Immunol (2017) 8:1875. 10.3389/fimmu.2017.01875 29312348 PMC5742132

[105] Hussen J Frank C Duvel A Koy M Schuberth HJ The chemokine CCL5 induces selective migration of bovine classical monocytes and drives their differentiation into LPS-hyporesponsive macrophages in vitro. Dev Comp Immunol (2014) 47:169–77. 10.1016/j.dci.2014.07.014 25064684

[106] Hussen J Duvel A Sandra O Smith D Sheldon IM Zieger P Phenotypic and functional heterogeneity of bovine blood monocytes. PloS One (2013) 8:e71502. 10.1371/journal.pone.0071502 23967219 PMC3743816

[107] Payne NR Frestedt J Hunkeler N Gehrz R Cell-surface expression of immunoglobulin G receptors on the polymorphonuclear leukocytes and monocytes of extremely premature infants. Pediatr Res (1993) 33:452–7. 10.1203/00006450-199305000-00007 7685516

[108] Hu JM Liu K Liu JH Jiang XL Wang XL Chen YZ CD163 as a marker of M2 macrophage, contribute to predicte aggressiveness and prognosis of Kazakh esophageal squamous cell carcinoma. Oncotarget (2017) 8:21526–38. 10.18632/oncotarget.15630 PMC5400603 28423526

[109] Abeles RD McPhail MJ Sowter D Antoniades CG Vergis N Vijay GK CD14, CD16 and HLA-DR reliably identifies human monocytes and their subsets in the context of pathologically reduced HLA-DR expression by CD14(hi) /CD16(neg) monocytes: Expansion of CD14(hi) /CD16(pos) and contraction of CD14(lo) /CD16(pos) monocytes in acute liver failure. Cytometry A (2012) 81:823–34. 10.1002/cyto.a.22104 22837127

[110] Moreno S Alvarez B Poderoso T Revilla C Ezquerra A Alonso F Porcine monocyte subsets differ in the expression of CCR2 and in their responsiveness to CCL2. Vet Res (2010) 41:76. 10.1051/vetres/2010048 20670605 PMC2941139

[111] Chamorro S Revilla C Alvarez B Alonso F Ezquerra A Dominguez J Phenotypic and functional heterogeneity of porcine blood monocytes and its relation with maturation. Immunology (2005) 114:63–71. 10.1111/j.1365-2567.2004.01994.x 15606796 PMC1782062

[112] Corripio-Miyar Y Hope J McInnes CJ Wattegedera SR Jensen K Pang Y Phenotypic and functional analysis of monocyte populations in cattle peripheral blood identifies a subset with high endocytic and allogeneic T-cell stimulatory capacity. Vet Res (2015) 46:112. 10.1186/s13567-015-0246-4 26407849 PMC4582714

[113] Ziegler-Heitbrock L Monocyte subsets in man and other species. Cell Immunol (2014) 289:135–9. 10.1016/j.cellimm.2014.03.019 24791698

[114] Gordon S Taylor PR Monocyte and macrophage heterogeneity. Nat Rev Immunol (2005) 5:953–64. 10.1038/nri1733 16322748

[115] Ziegler-Heitbrock L Ancuta P Crowe S Dalod M Grau V Hart DN Nomenclature of monocytes and dendritic cells in blood. Blood (2010) 116:e74–80. 10.1182/blood-2010-02-258558 20628149

[116] Ingersoll MA Spanbroek R Lottaz C Gautier EL Frankenberger M Hoffmann R Comparison of gene expression profiles between human and mouse monocyte subsets. Blood (2010) 115:e10–9. 10.1182/blood-2009-07-235028 PMC2810986 19965649

[117] Zawada AM Rogacev KS Schirmer SH Sester M Bohm M Fliser D Monocyte heterogeneity in human cardiovascular disease. Immunobiology (2012) 217:1273–84. 10.1016/j.imbio.2012.07.001 22898391

[118] Fairbairn L Kapetanovic R Beraldi D Sester DP Tuggle CK Archibald AL Comparative analysis of monocyte subsets in the pig. J Immunol (2013) 190:6389–96. 10.4049/jimmunol.1300365 23667115

[119] Gibbons N Goulart MR Chang YM Efstathiou K Purcell R Wu Y Phenotypic heterogeneity of peripheral monocytes in healthy dogs. Vet Immunol Immunopathol (2017) 190:26–30. 10.1016/j.vetimm.2017.06.007 28778319

[120] Haverkamp AK Lehmbecker A Spitzbarth I Widagdo W Haagmans BL Segales J Experimental infection of dromedaries with Middle East respiratory syndrome-Coronavirus is accompanied by massive ciliary loss and depletion of the cell surface receptor dipeptidyl peptidase 4. Sci Rep (2018) 8:9778. 10.1038/s41598-018-28109-2 29950581 PMC6021449

[121] Pomeroy B Sipka A Hussen J Eger M Schukken Y Schuberth HJ Counts of bovine monocyte subsets prior to calving are predictive for postpartum occurrence of mastitis and metritis. Vet Res (2017) 48:13. 10.1186/s13567-017-0415-8 28222802 PMC5320682

[122] Ozanska A Szymczak D Rybka J Pattern of human monocyte subpopulations in health and disease. Scand J Immunol (2020) 92:e12883. 10.1111/sji.12883 32243617

[123] Wong KL Yeap WH Tai JJ Ong SM Dang TM Wong SC The three human monocyte subsets: implications for health and disease. Immunol Res (2012) 53:41–57. 10.1007/s12026-012-8297-3 22430559

[124] Stansfield BK Ingram DA Clinical significance of monocyte heterogeneity. Clin Trans Med (2015) 4:5. 10.1186/s40169-014-0040-3 PMC4384980 25852821

[125] Beetz S Wesch D Marischen L Welte S Oberg HH Kabelitz D Innate immune functions of human gammadelta T cells. Immunobiology (2008) 213:173–82. 10.1016/j.imbio.2007.10.006 18406365

[126] Byers SR Evermann JF Bradway DS Grimm AL Ridpath JF Parish SM The effects of exposure of susceptible alpacas to alpacas persistently infected with bovine viral diarrhea virus. Can Vet J (2011) 52:263–71. PMC3039895 21629418

[127] Topliff CL Alkheraif AA Kuszynski CA Davis WC Steffen DJ Schmitz JA Experimental acute infection of alpacas with Bovine viral diarrhea virus 1 subgenotype b alters peripheral blood and GALT leukocyte subsets. J Vet Diagn Invest (2017) 29:186–92. 10.1177/1040638717690015 28166712

[128] Nourshargh S Alon R Leukocyte migration into inflamed tissues. Immunity (2014) 41:694–707. 10.1016/j.immuni.2014.10.008 25517612

[129] Waters WR Rahner TE Palmer MV Cheng D Nonnecke BJ Whipple DL Expression of L-Selectin (CD62L), CD44, and CD25 on activated bovine T cells. Infect Immun (2003) 71:317–26. 10.1128/IAI.71.1.317-326.2003 PMC143305 12496181

[130] Berard M Tough DF Qualitative differences between naive and memory T cells. Immunology (2002) 106:127–38. 10.1046/j.1365-2567.2002.01447.x PMC1782715 12047742

[131] Sallusto F Geginat J Lanzavecchia A Central memory and effector memory T cell subsets: function, generation, and maintenance. Annu Rev Immunol (2004) 22:745–63. 10.1146/annurev.immunol.22.012703.104702 15032595

[132] Blunt L Hogarth PJ Kaveh DA Webb P Villarreal-Ramos B Vordermeier HM Phenotypic characterization of bovine memory cells responding to mycobacteria in IFNgamma enzyme linked immunospot assays. Vaccine (2015) 33:7276–82. 10.1016/j.vaccine.2015.10.113 26549366

[133] Maggioli MF Palmer MV Thacker TC Vordermeier HM Waters WR Characterization of effector and memory T cell subsets in the immune response to bovine tuberculosis in cattle. PloS One (2015) 10:e0122571. 10.1371/journal.pone.0122571 25879774 PMC4400046

[134] Foote MR Nonnecke BJ Fowler MA Miller BL Beitz DC Waters WR Effects of age and nutrition on expression of CD25, CD44, and L-selectin (CD62L) on T-cells from neonatal calves. J Dairy Sci (2005) 88:2718–29. 10.3168/jds.S0022-0302(05)72951-9 16027185

[135] Kohlmeier JE Chan MA Benedict SH Costimulation of naive human CD4 T cells through intercellular adhesion molecule-1 promotes differentiation to a memory phenotype that is not strictly the result of multiple rounds of cell division. Immunology (2006) 118:549–58. 10.1111/j.1365-2567.2006.02396.x PMC1782322 16895560

[136] Spellberg B Edwards JE Jr. Type 1/Type 2 immunity in infectious diseases. Clin Infect Dis (2001) 32:76–102. 10.1086/317537 11118387

[137] Odbileg R Purevtseren B Batsukh Z Konnai S Ohashi K Onuma M Complete cDNA sequences and phylogenetic analyses of the Th1 and Th2 cytokines of the bactrian camel (Camelus bactrianus). J Vet Med Sci (2006) 68:941–6. 10.1292/jvms.68.941 17019063

[138] Odbileg R Purevtseren B Gantsetseg D Boldbaatar B Buyannemekh T Galmandakh Z Cytokine responses in camels (Camelus bactrianus) vaccinated with Brucella abortus strain 19 vaccine. J Vet Med Sci (2008) 70:197–201. 10.1292/jvms.70.197 18319583

[139] Yu X Wu Y Zhang J Jirimutu Zulipikaer A Chen J Pre-evaluation of humoral immune response of Bactrian camels by the quantification of Th2 cytokines using real-time PCR. J Biomed Res (2020) 34:387–94. 10.7555/JBR.34.20190035 PMC7540241 32611846

[140] Dorneles EM Lima GK Teixeira-Carvalho A Araujo MS Martins-Filho OA Sriranganathan N Immune Response of Calves Vaccinated with Brucella abortus S19 or RB51 and Revaccinated with RB51. PloS One (2015) 10:e0136696. 10.1371/journal.pone.0136696 26352261 PMC4564183

[141] Brown WC Rice-Ficht AC Estes DM Bovine type 1 and type 2 responses. Vet Immunol Immunopathol (1998) 63:45–55. 10.1016/S0165-2427(98)00081-6 9656440

[142] Osman R Gonzalez-Cano P Brownlie R Griebel PJ Induction of interferon and interferon-induced antiviral effector genes following a primary bovine herpesvirus-1 (BHV-1) respiratory infection. J Gen Virol (2017) 98:1831–42. 10.1099/jgv.0.000825 28675355

[143] Lopez de Padilla CM Niewold TB The type I interferons: Basic concepts and clinical relevance in immune-mediated inflammatory diseases. Gene (2016) 576:14–21. 10.1016/j.gene.2015.09.058 26410416 PMC4666791

[144] Premraj A Aleyas AG Nautiyal B Rasool TJ Camelid type I interferons: Identification and functional characterization of interferon alpha from the dromedary camel (Camelus dromedarius). Mol Immunol (2020) 119:132–43. 10.1016/j.molimm.2020.01.020 PMC7112685 32014632

[145] Abdel-Fattah M Saeed H El-Shennawy L Shalaby M Embaby A Ataya F The Arabian camel, Camelus dromedarius interferon epsilon: Functional expression, in vitro refolding, purification and cytotoxicity on breast cancer cell lines. PloS One (2019) 14:e0213880. 10.1371/journal.pone.0213880 31490936 PMC6730848

[146] Wernery U Kaaden OR Foot-and-mouth disease in camelids: a review. Vet J (2004) 168:134–42. 10.1016/j.tvjl.2003.10.005 15301761

[147] Lefranc MP Immunoglobulin and T Cell Receptor Genes: IMGT((R)) and the Birth and Rise of Immunoinformatics. Front Immunol (2014) 5:22. 10.3389/fimmu.2014.00022 24600447 PMC3913909

[148] Ciccarese S Burger PA Ciani E Castelli V Linguiti G Plasil M The Camel Adaptive Immune Receptors Repertoire as a Singular Example of Structural and Functional Genomics. Front Genet (2019) 10:997. 10.3389/fgene.2019.00997 31681428 PMC6812646

[149] Kotsias F Cebrian I Alloatti A Antigen processing and presentation. Int Rev Cell Mol Biol (2019) 348:69–121. 10.1016/bs.ircmb.2019.07.005 31810556

[150] Kelly A Trowsdale J Genetics of antigen processing and presentation. Immunogenetics (2019) 71:161–70. 10.1007/s00251-018-1082-2 PMC6394470 30215098

[151] Parham P Moffett A Variable NK cell receptors and their MHC class I ligands in immunity, reproduction and human evolution. Nat Rev Immunol (2013) 13:133–44. 10.1038/nri3370 PMC3956658 23334245

[152] Aluvihare VR Kallikourdis M Betz AG Regulatory T cells mediate maternal tolerance to the fetus. Nat Immunol (2004) 5:266–71. 10.1038/ni1037 14758358

[153] Somerset DA Zheng Y Kilby MD Sansom DM Drayson MT Normal human pregnancy is associated with an elevation in the immune suppressive CD25+ CD4+ regulatory T-cell subset. Immunology (2004) 112:38–43. 10.1111/j.1365-2567.2004.01869.x 15096182 PMC1782465

[154] Eger M Hussen J Drong C Meyer U von Soosten D Frahm J Impacts of parturition and body condition score on glucose uptake capacity of bovine monocyte subsets. Vet Immunol Immunopathol (2015) 166:33–42. 10.1016/j.vetimm.2015.04.007 25980551

[155] Ott TL Gifford CA Effects of early conceptus signals on circulating immune cells: lessons from domestic ruminants. Am J Reprod Immunol (2010) 64:245–54. 10.1111/j.1600-0897.2010.00912.x 20738264

[156] Kamat MM Vasudevan S Maalouf SA Townson DH Pate JL Ott TL Changes in Myeloid Lineage Cells in the Uterus and Peripheral Blood of Dairy Heifers During Early Pregnancy. Biol Reprod (2016) 95:68. 10.1095/biolreprod.116.141069 27512154

[157] Spadaro M Martire S Marozio L Mastromauro D Montanari E Perga S Immunomodulatory Effect of Pregnancy on Leukocyte Populations in Patients With Multiple Sclerosis: A Comparison of Peripheral Blood and Decidual Placental Tissue. Front Immunol (2019) 10:1935. 10.3389/fimmu.2019.01935 31474999 PMC6707093

[158] Oliveira LJ Barreto RS Perecin F Mansouri-Attia N Pereira FT Meirelles FV Modulation of maternal immune system during pregnancy in the cow. Reprod Domest Anim = Zuchthygiene (2012) 47 Suppl 4:384–93. 10.1111/j.1439-0531.2012.02102.x 22827396

[159] Leung ST Derecka K Mann GE Flint AP Wathes DC Uterine lymphocyte distribution and interleukin expression during early pregnancy in cows. J Reprod Fertil (2000) 119:25–33. 10.1530/jrf.0.1190025 10864810

[160] Piccione G Rizzo M Arfuso F Giannetto C Di Pietro S Bazzano M Leukocyte modifications during the first month after foaling in mares and their newborn foals. Pol J Vet Sci (2015) 18:621–5. 10.1515/pjvs-2015-0080 26618596

[161] Bazzano M Giannetto C Fazio F Rizzo M Giudice E Piccione G Physiological adjustments of haematological profile during the last trimester of pregnancy and the early post partum period in mares. Anim Reprod Sci (2014) 149:199–203. 10.1016/j.anireprosci.2014.07.005 25064559

[162] Zhang J Shynlova O Sabra S Bang A Briollais L Lye SJ Immunophenotyping and activation status of maternal peripheral blood leukocytes during pregnancy and labour, both term and preterm. J Cell Mol Med (2017) 21:2386–402. 10.1111/jcmm.13160 PMC5618694 28429508

[163] Nagamatsu T Schust DJ The immunomodulatory roles of macrophages at the maternal-fetal interface. Reprod Sci (2010) 17:209–18. 10.1177/1933719109349962 20065301

[164] Lash GE Robson SC Bulmer JN Review: Functional role of uterine natural killer (uNK) cells in human early pregnancy decidua. Placenta (2010) 31 Suppl:S87–92. 10.1016/j.placenta.2009.12.022 20061017

[165] Groebner AE Schulke K Schefold JC Fusch G Sinowatz F Reichenbach HD Immunological mechanisms to establish embryo tolerance in early bovine pregnancy. Reprod Fertil Dev (2011) 23:619–32. 10.1071/RD10230 21635810

[166] Nazifi S Ahmadi MR Gheisari HR Hematological changes of dairy cows in postpartum period and early pregnancy. Comp Clin Pathol (2008) 17:157–63. 10.1007/s00580-008-0730-6

[167] Tan EK Tan EL Alterations in physiology and anatomy during pregnancy. Best Pract Res Clin Obstet Gynaecol (2013) 27:791–802. 10.1016/j.bpobgyn.2013.08.001 24012425

[168] Hine BC Cartwright SL Mallard BA Analysis of leukocyte populations in Canadian Holsteins classified as high or low immune responders for antibody- or cell-mediated immune response. Can J Vet Res (2012) 76:149–56. PMC3314438 23024458

[169] Barriga C Rodriguez AB Ortega E Increased phagocytic activity of polymorphonuclear leukocytes during pregnancy. Eur J Obstet Gynecol Reprod Biol (1994) 57:43–6. 10.1016/0028-2243(94)90109-0 7821502

[170] Sheldon IM Cronin J Goetze L Donofrio G Schuberth HJ Defining postpartum uterine disease and the mechanisms of infection and immunity in the female reproductive tract in cattle. Biol Reprod (2009) 81:1025–32. 10.1095/biolreprod.109.077370 PMC2784443 19439727

[171] Saad AM Concha C Astrom G Alterations in neutrophil phagocytosis and lymphocyte blastogenesis in dairy cows around parturition. Zentralbl Veterinarmed B (1989) 36:337–45. 10.1111/j.1439-0450.1989.tb00612.x 2781892

[172] Ghazi SR Oryan A Pourmirzaei H Some aspects of macroscopic studies of the placentation in the camel (Camelus dromedarius). Anat Histol Embryol (1994) 23:337–42. 10.1111/j.1439-0264.1994.tb00483.x 7887485

[173] Furukawa S Kuroda Y Sugiyama A A comparison of the histological structure of the placenta in experimental animals. J Toxicol Pathol (2014) 27:11–8. 10.1293/tox.2013-0060 PMC4000068 24791062

[174] Tizard I The protective properties of milk and colostrum in non-human species. Adv Nutr Res (2001) 10:139–66. 10.1007/978-1-4615-0661-4_7 11795038

[175] Baintner K Transmission of antibodies from mother to young: Evolutionary strategies in a proteolytic environment. Vet Immunol Immunopathol (2007) 117:153–61. 10.1016/j.vetimm.2007.03.001 17459489

[176] Kamber R Farah Z Rusch P Hassig M Studies on the supply of immunoglobulin G to newborn camel calves (Camelus dromedarius). J Dairy Res (2001) 68:1–7. 10.1017/S0022029900004635 11289259

[177] Hülsebusch CG Immunoglobulin -G status of camels during six months post-natum. Rev Élev. Méd. vét. Pays Trop (2000) 53:105–10. 10.19182/remvt.9730

[178] Salhi I Bessalah S Mbarek SB Chniter M Seddik MM Khorchani T Passive transfer of maternal immunity in the dromedary (Camelus dromedarius), involvement of heavy chain antibodies. Trop Anim Health Prod (2015) 47:613–8. 10.1007/s11250-014-0751-x 25547806

[179] Meyer B Juhasz J Barua R Das Gupta A Hakimuddin F Corman VM Time Course of MERS-CoV Infection and Immunity in Dromedary Camels. Emerg Infect Dis (2016) 22:2171–3. 10.3201/eid2212.160382 PMC5189137 27224315

[180] Azwai SM Carter SD Woldehiwet Z The isolation and characterization of camel (Camelus dromedarius) immunoglobulin classes and subclasses. J Comp Pathol (1993) 109:187–95. 10.1016/S0021-9975(08)80262-9 8245233

[181] Azwai SM Carter SD Woldehiwet Z Monoclonal antibodies against camel (Camelus dromedarius) IgG, IgM and light chains. Vet Immunol Immunopathol (1995) 45:175–84. 10.1016/0165-2427(94)05334-O 7604534

[182] Azwai SM Carter SD Woldehiwet Z Immunoglobulins of camel (Camelus dromedarius) colostrum. J Comp Pathol (1996) 114:273–82. 10.1016/S0021-9975(96)80049-1 8762585

[183] Hamers-Casterman C Atarhouch T Muyldermans S Robinson G Hamers C Songa EB Naturally occurring antibodies devoid of light chains. Nature (1993) 363:446–8. 10.1038/363446a0 8502296

[184] El Sheikh AI Almathen F Hussen J Investigation of total immunoglobulin G concentration, heavy chain antibody levels, and neutrophil to lymphocyte ratio in female camels and their newborn calves. Trop Anim Health Prod (2020) 52(6):3863–8. 10.1007/s11250-020-02425-0 32996038

[185] Donovan DC Reber AJ Gabbard JD Aceves-Avila M Galland KL Holbert KA Effect of maternal cells transferred with colostrum on cellular responses to pathogen antigens in neonatal calves. Am J Vet Res (2007) 68:778–82. 10.2460/ajvr.68.7.778 17605614

[186] Bandrick M Ariza-Nieto C Baidoo SK Molitor TW Colostral antibody-mediated and cell-mediated immunity contributes to innate and antigen-specific immunity in piglets. Dev Comp Immunol (2014) 43:114–20. 10.1016/j.dci.2013.11.005 PMC3902642 24252519

[187] Langel SN Wark WA Garst SN James RE McGilliard ML Petersson-Wolfe CS Effect of feeding whole compared with cell-free colostrum on calf immune status: The neonatal period. J Dairy Sci (2015) 98:3729–40. 10.3168/jds.2014-8422 25795487

[188] Langel SN Wark WA Garst SN James RE McGilliard ML Petersson-Wolfe CS Effect of feeding whole compared with cell-free colostrum on calf immune status: Vaccination response. J Dairy Sci (2016) 99:3979–94. 10.3168/jds.2015-9892 26923041

[189] Meganck V Opsomer G Piepers S Cox E Goddeeris BM Maternal colostral leukocytes appear to enhance cell-mediated recall response, but inhibit humoral recall response in prime-boost vaccinated calves. J Reprod Immunol (2016) 113:68–75. 10.1016/j.jri.2015.11.004 26796988

[190] Cacho NT Lawrence RM Innate Immunity and Breast Milk. Front Immunol (2017) 8:584. 10.3389/fimmu.2017.00584 28611768 PMC5447027

[191] Elghetany MT Lacombe F Physiologic variations in granulocytic surface antigen expression: impact of age, gender, pregnancy, race, and stress. J Leukoc Biol (2004) 75:157–62. 10.1189/jlb.0503245 14557386

[192] Romanyukha AA Yashin AI Age related changes in population of peripheral T cells: towards a model of immunosenescence. Mech Ageing Dev (2003) 124:433–43. 10.1016/S0047-6374(03)00019-8 12714250

[193] Kampen AH Olsen I Tollersrud T Storset AK Lund A Lymphocyte subpopulations and neutrophil function in calves during the first 6 months of life. Vet Immunol Immunopathol (2006) 113:53–63. 10.1016/j.vetimm.2006.04.001 16772096

[194] Ayoub IA Yang TJ Age-dependent changes in peripheral blood lymphocyte subpopulations in cattle: a longitudinal study. Dev Comp Immunol (1996) 20:353–63. 10.1016/S0145-305X(96)00024-9 9016389

[195] Platt R Sponseller BA Chiang YW Roth JA Cell-mediated immunity evaluation in foals infected with virulent equine herpesvirus-1 by multi-parameter flow cytometry. Vet Immunol Immunopathol (2010) 135:275–81. 10.1016/j.vetimm.2009.12.010 20116862

[196] Cancela CSP Murao M Assumpcao JG Souza MEL de Macedo AV Viana MB Immunophenotyping of the cerebrospinal fluid as a prognostic factor at diagnosis of acute lymphoblastic leukemia in children and adolescents. Pediatr Hematol Oncol (2017) 34:53–65. 10.1080/08880018.2017.1313920 28548878

[197] Voiculescu C Avramescu C Radu E Voinea I [The importance of lymphocytic immunophenotyping in the clinical and therapeutic monitoring of acute joint rheumatism in school-aged children]. Bacteriol Virusol Parazitol Epidemiol (1997) 42:229–35. 9586333

[198] McCloskey TW Cavaliere T Bakshi S Harper R Fagin J Kohn N Immunophenotyping of T lymphocytes by three-color flow cytometry in healthy newborns, children, and adults. Clin Immunol Immunopathol (1997) 84:46–55. 10.1006/clin.1997.4370 9191883

[199] de Mendonca Picinin IF Camargos P Mascarenhas RF Santos SM Marguet C Cell count and lymphocyte immunophenotyping of bronchoalveolar lavage fluid in healthy Brazilian children. Eur Respir J (2011) 38:738–9. 10.1183/09031936.00006711 21885421

[200] Bailey M The mucosal immune system: recent developments and future directions in the pig. Dev Comp Immunol (2009) 33:375–83. 10.1016/j.dci.2008.07.003 18760299

[201] Gaashan MM Al-Mubarak AIA Hussen J Leukocyte populations and their cell adhesion molecules expression in newborn dromedary camel calves. Vet World (2020) 13:1863–9. 10.14202/vetworld.2020.1863-1869 PMC7566236 33132598

[202] O’Connell EM Nutman TB Eosinophilia in Infectious Diseases. Immunol Allergy Clin North Am (2015) 35:493–522. 10.1016/j.iac.2015.05.003 26209897 PMC4515572

[203] Guzman E Hope J Taylor G Smith AL Cubillos-Zapata C Charleston B Bovine gammadelta T cells are a major regulatory T cell subset. J Immunol (2014) 193:208–22. 10.4049/jimmunol.1303398 PMC4065783 24890724

[204] Nicholson GC Tennant RC Carpenter DC Sarau HM Kon OM Barnes PJ A novel flow cytometric assay of human whole blood neutrophil and monocyte CD11b levels: upregulation by chemokines is related to receptor expression, comparison with neutrophil shape change, and effects of a chemokine receptor (CXCR2) antagonist. Pulm Pharmacol Ther (2007) 20:52–9. 10.1016/j.pupt.2005.11.009 16406722

[205] Linssen J Aderhold S Nierhaus A Frings D Kaltschmidt C Zanker K Automation and validation of a rapid method to assess neutrophil and monocyte activation by routine fluorescence flow cytometry in vitro. Cytometry B Clin Cytom (2008) 74:295–309. 10.1002/cyto.b.20422 18431775

[206] Randall TD Mebius RE The development and function of mucosal lymphoid tissues: a balancing act with micro-organisms. Mucosal Immunol (2014) 7:455–66. 10.1038/mi.2014.11 24569801

[207] Wang WH Observations on aggregated lymphoid nodules in the cardiac glandular areas of the Bactrian camel (Camelus bactrianus). Vet J (2003) 166:205–9. 10.1016/S1090-0233(02)00263-0 12902188

[208] Xu XH Wang WH Gao Q Qi SS He WH Tai LF The anatomical characteristics of the aggregated lymphoid nodule area in the stomach of Bactrian camels (Camelus bactrianus) of different ages. Vet J (2010) 184:362–5. 10.1016/j.tvjl.2009.03.003 19375958

[209] ZhaXi Y Wang W Zhang W Gao Q Guo M Jia S Morphologic observation of mucosa-associated lymphoid tissue in the large intestine of Bactrian camels (Camelus bactrianus). Anat Rec (Hoboken) (2014) 297:1292–301. 10.1002/ar.22939 24820911

[210] Zhang WD Wang WH Jia S Distribution of immunoglobulin G antibody secretory cells in small intestine of Bactrian camels (Camelus bactrianus). BMC Vet Res (2015) 11:222. 10.1186/s12917-015-0538-y 26303329 PMC4547423

[211] Alluwaimi AM Fath El-Bab MR Ahemed AK Ali AMA Studies on the ileal lymphoid tissue (Peyer’s patches) in camels, Najdi sheep and cattle. J Camel Pract Res (1998) 5:13–8.

[212] Ehrlich SD The human gut microbiome impacts health and disease. C R Biol (2016) 339:319–23. 10.1016/j.crvi.2016.04.008 27236827

[213] Bull MJ Plummer NT Part 1: The Human Gut Microbiome in Health and Disease. Integr Med (Encinitas) (2014) 13:17–22. 26770121 PMC4566439

[214] Qi SS Wang WH Gao Q Xu XH He WH Zhaxi YP Age-related changes in the anatomical characteristics of Peyer’s patches in small intestine of Bactrian camels (Camelus bactrianus). Trop Anim Health Prod (2011) 43:1219–23. 10.1007/s11250-011-9829-x 21461871

[215] Zidan M Pabst R Unique microanatomy of ileal Peyer’s patches of the one humped camel (Camelus dromedarius) is not age-dependent. Anatomical Record (2008) 291:1023–8. 10.1002/ar.20697 18449903

[216] Cerutti A Chen K Chorny A Immunoglobulin responses at the mucosal interface. Annu Rev Immunol (2011) 29:273–93. 10.1146/annurev-immunol-031210-101317 PMC3064559 21219173

[217] Zhang WD Wang WH Jia S The Distribution of SIgA and IgG Antibody-Secreting Cells in the Small Intestine of Bactrian Camels (Camelus bactrianus) of Different Ages. PloS One (2016) 11:e0156635. 10.1371/journal.pone.0156635 27249417 PMC4889134

[218] Gassen NC Niemeyer D Muth D Corman VM Martinelli S Gassen A SKP2 attenuates autophagy through Beclin1-ubiquitination and its inhibition reduces MERS-Coronavirus infection. Nat Commun (2019) 10:5770. 10.1038/s41467-019-13659-4 31852899 PMC6920372

[219] Inn KS Kim Y Aigerim A Park U Hwang ES Choi MS Reduction of soluble dipeptidyl peptidase 4 levels in plasma of patients infected with Middle East respiratory syndrome coronavirus. Virology (2018) 518:324–7. 10.1016/j.virol.2018.03.015 PMC7112025 29587190

[220] Zaki AM van Boheemen S Bestebroer TM Osterhaus AD Fouchier RA Isolation of a novel coronavirus from a man with pneumonia in Saudi Arabia. N Engl J Med (2012) 367:1814–20. 10.1056/NEJMoa1211721 23075143

[221] Mok CKP Zhu A Zhao J Lau EHY Wang J Chen Z T-cell responses to MERS coronavirus infection in people with occupational exposure to dromedary camels in Nigeria: an observational cohort study. Lancet Infect Dis (2020). 10.1016/S1473-3099(20)30599-5 PMC7538089 33035474

[222] Adney DR van Doremalen N Brown VR Bushmaker T Scott D de Wit E Replication and shedding of MERS-CoV in upper respiratory tract of inoculated dromedary camels. Emerg Infect Dis (2014) 20:1999–2005. 10.3201/eid2012.141280 25418529 PMC4257817

[223] Haagmans BL van den Brand JM Raj VS Volz A Wohlsein P Smits SL An orthopoxvirus-based vaccine reduces virus excretion after MERS-CoV infection in dromedary camels. Science (2016) 351:77–81. 10.1126/science.aad1283 26678878

[224] Reusken CB Haagmans BL Muller MA Gutierrez C Godeke GJ Meyer B Middle East respiratory syndrome coronavirus neutralising serum antibodies in dromedary camels: a comparative serological study. Lancet Infect Dis (2013) 13:859–66. 10.1016/S1473-3099(13)70164-6 PMC7106530 23933067

[225] Harcourt JL Rudoler N Tamin A Leshem E Rasis M Giladi M The prevalence of Middle East respiratory syndrome coronavirus (MERS-CoV) antibodies in dromedary camels in Israel. Zoonoses Public Health (2018) 65:749–54. 10.1111/zph.12482 PMC6274617 29855166

[226] Gossner C Danielson N Gervelmeyer A Berthe F Faye B Kaasik Aaslav K Human-Dromedary Camel Interactions and the Risk of Acquiring Zoonotic Middle East Respiratory Syndrome Coronavirus Infection. Zoonoses Public Health (2016) 63:1–9. 10.1111/zph.12171 25545147 PMC7165574

[227] Reusken C Haagmans BL Koopmans MP [Dromedary camels and Middle East respiratory syndrome: MERS coronavirus in the ‘ship of the desert’]. Ned Tijdschr Geneeskd (2014) 158:A7806. 25248734

[228] Reusken CB Farag EA Jonges M Godeke GJ El-Sayed AM Pas SD Middle East respiratory syndrome coronavirus (MERS-CoV) RNA and neutralising antibodies in milk collected according to local customs from dromedary camels, Qatar, April 2014. Euro Surveill (2014) 19:20829. 10.2807/1560-7917.ES2014.19.23.20829 24957745

[229] Lambeir AM Durinx C Scharpe S De Meester I Dipeptidyl-peptidase IV from bench to bedside: an update on structural properties, functions, and clinical aspects of the enzyme DPP IV. Crit Rev Clin Lab Sci (2003) 40:209–94. 10.1080/713609354 12892317

[230] Raj VS Mou H Smits SL Dekkers DH Muller MA Dijkman R Dipeptidyl peptidase 4 is a functional receptor for the emerging human coronavirus-EMC. Nature (2013) 495:251–4. 10.1038/nature12005 PMC7095326 23486063

[231] Ohnuma K Haagmans BL Hatano R Raj VS Mou H Iwata S Inhibition of Middle East respiratory syndrome coronavirus infection by anti-CD26 monoclonal antibody. J Virol (2013) 87:13892–9. 10.1128/JVI.02448-13 PMC3838260 24067970

[232] van Doremalen N Miazgowicz KL Milne-Price S Bushmaker T Robertson S Scott D Host species restriction of Middle East respiratory syndrome coronavirus through its receptor, dipeptidyl peptidase 4. J Virol (2014) 88:9220–32. 10.1128/JVI.00676-14 PMC4136254 24899185

[233] Widagdo W Raj VS Schipper D Kolijn K van Leenders GJ Bosch BJ Differential Expression of the Middle East Respiratory Syndrome Coronavirus Receptor in the Upper Respiratory Tracts of Humans and Dromedary Camels. J Virol (2016) 90:4838–42. 10.1128/JVI.02994-15 PMC4836314 26889022

[234] Pierson DM Jones D Muzzafar T Kersh MJ Challagundla P Medeiros LJ Utility of CD26 in flow cytometric immunophenotyping of T-cell lymphomas in tissue and body fluid specimens. Cytometry B Clin Cytom (2008) 74:341–8. 10.1002/cyto.b.20431 18727078

[235] Min CK Cheon S Ha NY Sohn KM Kim Y Aigerim A Comparative and kinetic analysis of viral shedding and immunological responses in MERS patients representing a broad spectrum of disease severity. Sci Rep (2016) 6:25359. 10.1038/srep25359 27146253 PMC4857172

[236] Alharbi NK Vaccines against Middle East respiratory syndrome coronavirus for humans and camels. Rev Med Virol (2017) 27:e1917. 10.1002/rmv.1917 PMC7169231 27786402

[237] Muthumani K Falzarano D Reuschel EL Tingey C Flingai S Villarreal DO A synthetic consensus anti-spike protein DNA vaccine induces protective immunity against Middle East respiratory syndrome coronavirus in nonhuman primates. Sci Transl Med (2015) 7:301ra132. 10.1126/scitranslmed.aac7462 PMC4573558 26290414

[238] Corman VM Albarrak AM Omrani AS Albarrak MM Farah ME Almasri M Viral Shedding and Antibody Response in 37 Patients With Middle East Respiratory Syndrome Coronavirus Infection. Clin Infect Dis (2016) 62:477–83. 10.1093/cid/civ951 PMC7108065 26565003

[239] Zhao J Alshukairi AN Baharoon SA Ahmed WA Bokhari AA Nehdi AM Recovery from the Middle East respiratory syndrome is associated with antibody and T-cell responses. Sci Immunol (2017) 2:eaan5393. 10.1126/sciimmunol.aan5393 28778905 PMC5576145

